# Ethnopharmacological Applications Targeting Alcohol Abuse: Overview and Outlook

**DOI:** 10.3389/fphar.2019.01593

**Published:** 2020-02-14

**Authors:** Laxman Singh, Tanuj Joshi, Devesh Tewari, Javier Echeverría, Andrei Mocan, Archana N. Sah, Emil Parvanov, Nikolay T. Tzvetkov, Zheng Feei Ma, Yeong Yeh Lee, Piotr Poznański, Lukasz Huminiecki, Mariusz Sacharczuk, Artur Jóźwik, Jarosław O. Horbańczuk, Joanna Feder-Kubis, Atanas G. Atanasov

**Affiliations:** ^1^ Centre for Biodiversity Conservation & Management, G.B. Pant National Institute of Himalayan Environment & Sustainable Development, Almora, India; ^2^ Department of Pharmaceutical Sciences, Faculty of Technology, Kumaun University Bhimtal Campus, Nainital, India; ^3^ Department of Pharmacognosy, School of Pharmaceutical Sciences, Lovely Professional University, Phagwara, India; ^4^ Institute of Genetics and Animal Breeding of the Polish Academy of Sciences, Jastrzebiec, Poland; ^5^ Department of Environmental Sciences, Faculty of Chemistry and Biology, Universidad de Santiago de Chile, Santiago, Chile; ^6^ Department of Pharmaceutical Botany, “Iuliu Hațieganu” University of Medicine and Pharmacy, Cluj-Napoca, Romania; ^7^ Institute of Molecular Genetics, Academy of Sciences of the Czech Republic, Division BIOCEV, Prague, Czechia; ^8^ Institute of Molecular Biology “Roumen Tsanev”, Department of Biochemical Pharmacology and Drug Design, Bulgarian Academy of Sciences, Sofia, Bulgaria; ^9^ Department Global R&D, NTZ Lab Ltd., Sofia, Bulgaria; ^10^ Department of Public Health, Xi’an Jiaotong-Liverpool University, Suzhou, China; ^11^ School of Medical Sciences, Universiti Sains Malaysia, Kota Bharu, Malaysia; ^12^ Faculty of Chemistry, Wrocław University of Science and Technology, Wybrzeże Wyspiańskiego, Wrocław, Poland; ^13^ Department of Pharmacognosy, University of Vienna, Vienna, Austria; ^14^ Institute of Neurobiology, Bulgarian Academy of Sciences, Sofia, Bulgaria; ^15^ Ludwig Boltzmann Institute for Digital Health and Patient Safety, Medical University of Vienna, Vienna, Austria

**Keywords:** alcohol, binge drinking, alcoholism, drug abuse, fatty liver, natural products

## Abstract

Excessive alcohol consumption is the cause of several diseases and thus is of a major concern for society. Worldwide alcohol consumption has increased by many folds over the past decades. This urgently calls for intervention and relapse counteract measures. Modern pharmacological solutions induce complete alcohol self-restraint and prevent relapse, but they have many side effects. Natural products are most promising as they cause fewer adverse effects. Here we discuss in detail the medicinal plants used in various traditional/folklore medicine systems for targeting alcohol abuse. We also comprehensively describe preclinical and clinical studies done on some of these plants along with the possible mechanisms of action.

## Introduction

Excessive alcohol consumption in the age range group of 15–64 years is responsible for 1 out of 7 deaths and 1 out of 13 death cases in men and women, respectively, which involve drinking during pregnancy, binge drinking, heavy drinking, and underage drinking (Sacks et al., 2015; Baldassarre et al., 2018). High alcohol intake is leading to 5.9% of all lethal cases among people, and excessive alcohol consumption puts around 5.1% of disease burden on the world (Organization and Unit, 2014).

Alcohol addiction and abuse is a complex disorder associated with biological, emotional, and social factors, which often leads to health problems—such as liver cirrhosis, hypertension, coronary artery disease, central nervous system disorders, alcohol-induced cardiomyopathy, and gastrointestinal disorder—and deteriorates economic prosperity of the family. Worldwide alcohol consumption has increased many folds over the decades, and abuse and addiction have been on the rise. The liver—the primary site for alcohol metabolism—is the most affected organ: excessive alcohol consumption causes cell to die, leaving scar tissue in their place. Prolonged high intake results in cirrhosis, which makes detoxification difficult while the organ is susceptible to infection and inflammation.

The adverse effects of the conventional medications for treatment of mainly the liver diseases caused by acute or chronic alcohol consumption are the reason for search of new alternative ways of cure. Under this circumstances, natural products are attractive option for treatment of alcohol-induced liver impairment (Ao et al., 2009). At present there is no therapeutic cure for alcoholic liver disease (ALD); hence, the development of novel medicines that are effective against alcoholic injury is the need of the hour (Kim et al., 2014). The modern pharmacological approaches are believed to play a vital role in achieving complete alcohol self-restraint and prevent relapse, but have limited efficacy with high adverse effects (Addolorato et al., 2005).

As far as the terminology is concerned, current use of alcohol can be demarcated as drinking at least one drink during the past 30 days and binge drinking can be defined as consumption of five or over five drinks in a day. Studies on the current alcohol use showed that 51% of adults who are above 18 years and 56% of adults who are between the age group of 18 to 44 drink alcohol regularly (Schiller et al., 2012). Excessive drinking is seen among 30% of the existing drinkers (Naimi et al., 2003). In the United States, 92% of adult heavy drinkers show a pattern of binge drinking in the last 30 days (Town et al., 2006). Thus, to combat the threat of alcoholism in the society, natural products can play a key role, with advantage of no or minimal adverse effects (Xu et al., 2005).

Various *in vitro* experiments involving HepG2 cells when treated with ethanol produced a significant reduction in glutathione (GSH) levels (Gutierrez-Ruiz et al., 1999; Kaur et al., 2009) and the generation of reactive oxygen species (ROS) (Ogony et al., 2008). One example of a natural product studied in this cellular model is antroquinonol, a tetrahydroubiquinone derivative that occurs mainly in the mycelium of *Antrodia camphorata* (Angamuthu et al., 2019). Pretreatment with antroquinonol in a dose-dependent manner provides protection to HepG2 cells against cellular lipid peroxidation and hepatic enzyme generation induced by ethanol. Additionally, sustained depletion of GSH by ethanol was also reversed by antroquinonol pretreatment (Kumar et al., 2011). Other than this, antroquinonol pretreatment is also known to provide protection to hepatic cells against oxidative stress produced by ethanol. The possible mechanism of action involves upregulation of expression of nuclear factor erythroid 2–related factor 2 (Nrf-2) gene that in turn downstream antioxidant genes arbitrated through mitogen-activated kinase proteins (MAPs) (Kumar et al., 2011).

### Development and Course of Alcohol Abuse

Alcohol addiction and abuse steadily progresses with time. Various researchers have given diverse stages regarding the progression of alcoholism. These stages vary in numbers according to the point of view of different researchers. According to their definitions, first, second, third, and fourth stages of alcoholism have been categorized as pre-alcoholic, early alcoholic, middle alcoholic, and late alcoholic, respectively. In the pre-alcoholic stage, the person is involved with social drinking, which does not cause any significant problems. An organism exposed to a slow increase in the amount of alcohol intake may start developing tolerance towards it. The habit of drinking, which is believed to relieve anxiety, stress, pain, and similar problems, may be the first step in addiction forming. In stage two of alcoholism, the person feels a mixed feeling of development of discomfort due to absence of alcohol intake and a strong desire to get alcohol. The person at this stage lies about his/her drinking habits to family and friends. The alcoholic finds new methods to secretly consume alcohol. Tolerance towards alcohol gradually progresses in this stage. In stage three, the symptoms of alcoholism become visible to friends and family. The relationship of the alcoholic with his/her family and friends begins to deteriorate. The alcoholic develops symptoms of alcohol abuse like weight loss or weight gain, facial redness, sluggishness, stomach bloating, etc.

The alcoholic now begins to suffer from severe complications like liver cirrhosis, dementia, and other ailments, which may lead to the loss of employment. Round-the-clock consumption progressively becomes an addiction and reduces or dislodges other activities, deteriorates the well-being of the family, and estranges friends. At this stage an attempt to get out of the habit results in hallucinations, tremors, and similar phenomena. Help can be found in professional rehabilitation centers (https://www.alcohol.org accessed on 01.03.2019). Prolonged alcoholism leads to psychological and physiological alterations inside the body and—among others—negatively affects various neurotransmitters.

There are various mechanisms associated with alcohol abuse, addiction, and dependence. One important effect by which alcohol leads to drug addiction and subsequently to its abuse is its effect on neurotransmitters. In acute alcohol ingestion, alcohol up-regulates GABAergic transmission and interferes with transmission of glutamate. Thus, due to intake of alcohol, the balance between the inhibitory and excitatory inputs is disturbed in the brain. In chronic alcohol ingestion, the brain in its attempt to attain equilibrium in presence of alcohol undergoes neuro-adaptations and this leads to enhancement in the level of glutamate and decrease in the level of gamma-aminobutyric acid (GABA). The activities of other neurotransmitters like serotonin, dopamine, adenosine, and glycine are also altered by alcohol. Apart from this, it interacts with the opioid system, endo-cannabinoid system, and nicotinic cholinergic system as well as cholinergic transmission. It has been investigated by scientists that interactions of various molecules with the opioid receptor system can be the reason behind their addiction and dependence-producing effects. This is supported by the fact that drugs like morphine, which interact with the opioid receptor system, have considerable addiction and dependence causing potentials. It has also been pointed out that the reinforcing effect of alcohol might be due to its interaction with the opioid receptor system. Studies have also shown that there is a very strong correlation between alcohol addiction and genetics. Genetic factors can play an important role in favoring the development of addiction by determining what neurochemical changes would be produced on both acute as well as chronic ingestion of alcohol. Thus, alcohol may produce drug addiction and abuse through a variety of mechanisms (Pickens and Svikis, 1988; Nestler, 2004; Tripathi, 2013; Michalak and Biala, 2016).

### Current Conventional Pharmacotherapy of Alcohol Dependence

Alcohol addiction, abuse and dependence have become severe problems affecting the lives of many people throughout the world. Currently at many rehabilitation centers, multiple allopathic drugs are being used to treat alcohol addiction and its complications. Though many allopathic drugs have proven to be a useful aid to combat alcoholism, yet drugs alone are not a complete answer for treatment of alcoholism. Successful treatment of alcoholism requires a combination of both psychological therapy as well as pharmacotherapy. Many drugs like benzodiazepines (BDZs) and disulfiram have been used in the treatment of alcohol dependence for many years but they have their own merits and demerits. Sometimes they may not prove to be very effective in an alcoholic patient and they have a potential to cause a variety of adverse effects. For example, by using BDZs in alcohol dependence, the alcoholic might himself/herself get addicted to BDZs. Also, in some studies it has been found that BDZs are not very effective in reducing craving associated with alcohol consumption. Similarly, taking disulfiram precludes even small amounts of alcohol or else severe adverse reactions within the body are likely to occur. Thus, the main action of disulfiram becomes its biggest drawback (Addolorato et al., 2002; Tripathi, 2013).

Many conventional drugs are in use for treatment of emergencies associated with alcohol addiction. One such is BDZs, having sedative, anxiolytic, and hypnotic action. BDZs have been tried in patients suffering from acute alcohol intoxication and showing aggressive behavior. They have been used as a replacement of alcohol, as a measure to stop the onset of withdrawal reactions precipitated by discontinuation of alcohol. In certain alcoholic patients, the person suffering from anxiety is reported to develop alcohol dependence. This is backed by research highlighted blending genetic similarities in the predisposition to develop anxiety and alcoholism. Thus, the use of BDZs enables the alcoholic person to cope with anxiety and trauma and it also protects the health care professionals against the aggressive behavior of the alcoholics. However, continuous use of BDZs in acute alcohol intoxication draws serious implications such as the following: it can lead to hypotension, impairment in consciousness, and depression of respiration (Addolorato et al., 2002; Shpilenya et al., 2002; Tripathi, 2013). So, one has to be conscious prescribing such formulations unless fully acquainted with the case study.

Contrarily, metadoxine and flumazenil, a competitive antagonist of BDZs, have been tried to enhance the rate of recovery from unconsciousness alcoholic state (Addolorato et al., 2002). For treatment of alcohol withdrawal syndrome, gamma hydroxybutyric acid (GHB), chlordiazepoxide, diazepam, and baclofen have also been tried, which play a key role in diminishing symptoms associated with withdrawal reactions like agitation, depression, anxiety, etc. Similarly, drugs like tiapride, tioridazine, and haloperidol have been found to be useful in treatment of delirium tremens (Mayo-Smith, 1997; Addolorato et al., 1999; Gallimberti et al., 2000; Addolorato et al., 2002).

Relapse of alcoholism is a major problem associated with the efforts to leave alcohol. Many drugs that have anti-craving, aversion-causing, and anti-reward effects have been used. Disulfiram is used as an important aversion-causing drug. Unpleasant reactions are produced if a person on disulfiram takes even very small quantity of alcohol. This strengthens the resolution of the alcoholic to refrain from consuming alcohol. The fact of the matter is ethanol metabolism by enzymes alcohol dehydrogenase (ADH) and aldehyde dehydrogenase (ALDH2) converts this first into acetaldehyde and then into acetic acid derivative, respectively. Disulfiram here plays a key role by inhibiting the enzyme ALDH2 as a result of which acetaldehyde is not converted to acetic acid. Subsequently, upon alcohol consumption the levels of acetaldehyde are increased and acetaldehyde produces unpleasant reactions like headache, respiratory depression, diarrhea, vomiting, nausea, hypotension, flushing, etc. Treatment with disulfiram has its own drawbacks in that concurrent alcohol consumption triggers serious reactions as mentioned above. Awareness of this fact must be raised on the part of the patient. The use of disulfiram is strictly prohibited in conditions like cardiopathy, diabetes, nephropathy, pregnancy, and in patients having history of drug allergy and hypersensitivity. Various drugs have also been used to reduce craving with different mechanisms. Some drugs reduce the craving for alcohol by mimicking the action of alcohol, whereas other drugs decrease the pleasant sensation associated with alcohol consumption (anti-reward effect) (Addolorato et al., 2002; Tripathi, 2013).

Similarly, GHB acid is used to decrease craving associated with alcohol consumption. It shows alcohol mimetic action and acts by producing interference in the functioning of some neurotransmitter systems like the mesolimbic cortical system. It does so by producing changes in levels of serotonin, dopamine, and GABA in brain. The downside of using GHB is that patients may start craving for GHB itself. GHB carries abuse and dependence producing liability, though this incidence is low but still supervision is highly recommended in using GHB treatment on alcoholics (Addolorato et al., 1996; Addolorato et al., 1997; Gallimberti et al., 2000; Gessa et al., 2000; Addolorato et al., 2002). Another class of drug is baclofen, which acts as a GABA_B_ agonist that is conventionally used as a centrally acting muscle relaxant but now it has also found use in treatment of alcohol dependence. Baclofen leads to alcohol abstinence and decreases craving. An advantage of baclofen is that it does not carry abuse liability but still more studies are needed to evaluate the risk/benefit ratio of baclofen (Addolorato et al., 2000; Colombo et al., 2000; Addolorato et al., 2002).

Opioid receptor antagonist like naltrexone is also currently used drug for treatment of alcoholism. It is believed that the opioid system is involved with the compulsive and reinforcing effects of alcohol that lead to craving desire associated with alcohol use. Thus, naltrexone is effective in decreasing craving towards alcohol. The disadvantage of naltrexone is that naltrexone produces side effects like insomnia, headache, vomiting, etc., and these can become more pronounced if the patient continues to consume alcohol. Also it is contraindicated in hepatic insufficiency and acute hepatitis (Volpicelli et al., 1992; Croop et al., 1997; Addolorato et al., 2000; Tripathi, 2013). Likewise, acamprosate is a drug that decreases the activity of the excitatory components of the brain. It does so by affecting calcium ion channels. It decreases craving and leads to alcohol abstinence. In a study, acamprosate has not shown to be very effective in the treatment of alcohol dependence and many studies are still needed to establish its effect in treatment of alcoholism (Paille et al., 1995; Sass et al., 1996; Addolorato et al., 2002).

Selective serotonin reuptake inhibitors (SSRIs), like fluoxetine, etc., have also found application in treatment of mood disturbances in alcoholics and their mechanism in treatment of alcoholism has been attributed to both GABAergic and serotonergic actions. SSRIs are basically effective in alcoholics with mood disturbances and provide a relief in alcoholics with depressive symptoms. Drugs like citalopram and sertraline are more effective in alcohol dependence with a late onset. Buspirone, which acts as a partial agonist at 5-^HT^1_A_ receptor, is a helpful drug in alcoholics with anxiety (Gorelick and Paredes, 1992; Sellers et al., 1994; Kranzler et al., 1995; Malec et al., 1996; Tripathi, 2013).

Another class of drug is the metadoxine that helps in restoring neuropsychological behavior in alcoholics to normal. In patients it was found to decrease psychomotor agitation, compulsive desire, aggressiveness, and improved work efficiency, emotions, and relationships in alcoholics. Metadoxine improves ethanol metabolism by affecting the liver enzyme system. Also levels of adenosine triphosphate (ATP) and release of acetylcholine and GABA are increased in the brain.

In summary, it can be said overall that conventional pharmacotherapy helps in the treatment of alcohol dependence and addiction, yet it has its own set of disadvantages and harmful effects. Thus, newer and safer treatment of alcohol dependence is still miles away (Bono et al., 1991; Caballeria et al., 1998; Stefanini et al., 1999).

## Effects of Alcohol Consumption on Hepatic and Cardiovascular Systems

A major causative agent for chronic liver disease (CLD) in the USA and Europe (Younossi et al., 2011; Blachier et al., 2013) is alcoholism. In 2010, 14.5 million disability-adjusted life-years and approx. 0.5 million deaths around the world were caused by ALD (Rehm et al., 2013). Excessive use of alcohol leads to hepatic steatosis (O’Shea et al., 2010), but only a subset of patients are known to develop clinically significant liver disease, depending on various behavior factors, genetic predisposition, and comorbidities. Out of these, one of the most important ones is obesity (Raynard et al., 2002; Parker et al., 2018). Alcohol has not only had a direct harmful effect on liver but indirect effect on other organs also. Dangerous use of alcohol alters adipose tissue functions and causes liver-damaging effects and progression of ALD (Parker et al., 2018).

Reasons behind different types of liver diseases in general are alcohol abuse, toxic drugs, metabolic disorders, hepatitis virus types A, B, and C, and chemicals, among others (Friedman, 2003). One of the most common reasons behind liver diseases in north-western Europe, United States, and other parts of the world is alcoholism and this condition is associated with mortality rates of 5% to 6% (Morris et al., 2012). Heavy alcohol consumption for a longer duration leads to higher risk of development of liver diseases (Younossi, 1998). Binge drinking leads to development of acute alcoholic hepatitis and if the problem becomes excessive it can even become life threatening (De et al., 2009). Many studies indicate that the levels of ROS, cellular lipid peroxidation, nitric oxide (NO), hepatic enzymes, cytokines, alanine aminotransferase (ALT), aspartate aminotransferase (AST), and tumor necrosis factor-alpha (TNFα) are enhanced by excessive ingestion of alcohol and play a vital role in progression and etiology of alcohol-induced hepatic diseases (Gutierrez-Ruiz et al., 1999; Nah et al., 2005; Kumar et al., 2011). Due to excessive free radicals generation by alcohol consumption, there is spontaneous reduction in the glutathione levels as well (Gutierrez-Ruiz et al., 1999; Kumar et al., 2011). Raised accumulation of intracellular ROS in hepatic cells and oxidative stress are key effects of ethanol exposure and these effects lead to the hepatic diseases (Das and Vasudevan, 2007).

Acetaminophen (paracetamol) is implicated as the causative agent of 42% of all the cases of acute liver failure (ALF) in the USA (Larson et al., 2005). A high risk factor of ALF is associated with consumption of acetaminophen in elderly, alcoholics, and in those cases where there is an overdose with this drug (Sass and Shakil, 2005; Dart and Bailey, 2007; Rhodes et al., 2011). There are higher incidences of acetaminophen toxicity in case of chronic alcoholics, people suffering from malnutrition, and the elderly (Larson et al., 2005; Dart and Bailey, 2007).

Various mediators of alcohol metabolism directly or indirectly lead to liver injury. Cytochrome P450 2E1 (CYP2E1) is the major enzyme that metabolizes alcohol. CYP2E1 does not produce any damage to liver if the amount of alcohol ingested is not excessive. However, if the amount of alcohol consumed by a person is very high, CYP2E1 leads to the formation of ROS like hydrogen peroxide, superoxide anion radical, and highly reactive conjugated adducts (Lu and Cederbaum, 2008).

Pro-fibrogenic cytokines, such as platelet-derived growth factor-beta (PDGF-β), transforming growth factor-beta (TGF-β), and connective tissue growth factor (CTGF) are released by hepatic stellate cells (HSC) on activation (Pinzani and Marra, 2001; Parsons et al., 2007). Inhibition of HSC activation is the main goal for the treatment of hepatic injury induced by alcohol consumption (Wang et al., 2006).

Important immune and endocrine functions relating to adipose tissues can be also altered by alcohol. These effects further enhance the toxic effect of alcohol on liver. Release of non-esterified fatty acids (NEFAs) into the systemic circulation occurs as a result of enhanced lipolysis in adipose tissues (Parker et al., 2018). Steatosis, insulin resistance, and hepatic inflammation result due to uptake of NEFAs by the liver (Parker et al., 2018). Liver function is also altered by changes in the adipokines secretion by adipocytes. There are convincing studies providing evidence on the actions of leptin, which serves as a proinflammatory and profibrotic agent. At last, adipose tissue inflammation caused by alcohol or obesity fosters the pro-inflammatory cytokines release into circulation and this results in a direct damage to liver and also leads to liver tissue infiltration by immune cells (Parker et al., 2018).

Endocrine function of adipose tissues is changed by consumption of alcohol and this varies depending on its use pattern and also the existence and stage of ALD (Parker et al., 2018). Circulating adiponectin is increased by both moderate (Sierksma et al., 2004; Beulens et al., 2006; Beulens et al., 2008; Brien et al., 2011) and high levels of alcohol (Hillemacher et al., 2009). In patients with ALD, there is maintenance of elevated serum adiponectin levels (Tacke et al., 2005), and the higher the adiponectin levels are, the greater will be the severity of ALD (Kaser et al., 2005; Kasztelan-Szczerbinska et al., 2013). Studies done on human adipocytes by *in vitro* methods have given evidence that expression of adipocytes is modulated in presence of alcohol (Ajmera et al., 2017).

Light consumption (0.1–5 g per day) of alcohol has several benefits like alcohol in low levels reduces severity of metabolic syndrome (Sun et al., 2014) and risk of cardiovascular disease (Costanzo et al., 2010); however, high amount consumption of alcohol (> 26 g per day) enhances risk of cardiovascular mortality (Costanzo et al., 2010). A close link between non-alcoholic fatty liver disease (NAFLD) and metabolic syndrome increased the death of the patients. There is correlation between dangerous consumption of alcohol and individual components of metabolic syndrome in general (Fan et al., 2006; Bessembinders et al., 2011; Briasoulis et al., 2012) and this correlation is directly proportional (Parker et al., 2018).

Alcoholism is a commonly encountered problem and has a significant effect on adipose tissues. Normal function, structure, and distribution of adipose tissue are disturbed by harmful use of alcohol. Liver functions are altered directly or indirectly by alterations in adipose tissues and this in turn leads to ALD. Inflammatory changes in adipose tissue are immediately reversed by stopping consumption of alcohol, and experimental ALD is improved by drugs that restore the functions of ALD to normal (Parker et al., 2018).

Alterations in adipose tissue by alcoholism occur in a similar manner in obesity and NAFLD as well. Risk of morbidity and mortality related to liver problems is enhanced by synergism of obesity and alcohol consumption. There should be awareness among physicians who are treating patients with ALD regarding the consequences of adipose tissue dysfunctions affecting the functions of liver. They should also develop effective plans for management of insulin resistance and obesity. Knowledge into the extra hepatic actions of alcohol involvement in the development of ALD will help in development of efficient treatments (Parker et al., 2018).

## Alcohol and Nicotine: a Dangerous Combination

Worldwide nicotine and alcohol are often abused in combination as drugs and lead to deaths of 9 million people every year in a combined manner (Ostroumov et al., 2015). There is a strong evidence of positive correlation between use of nicotine and alcohol (DiFranza and Guerrera, 1990; Miller and Gold, 1998; Dani and Harris, 2005; Weitzman and Chen, 2005; Barrett et al., 2006). There is more vulnerability of binge drinking in both regular smokers and non-regular (non-dependent) smokers than non-smokers (Weitzman and Chen, 2005; Harrison et al., 2008; Campbell et al., 2012).

Some subjective rewarding alcohol effects are enhanced through nicotine and vice versa (Glautier et al., 1996; Kouri et al., 2004; Rose et al., 2004). In addition, nicotine can influence alcohol consumption in a longer run. Use of nicotine at a young age enhances the development of disorders associated with consumption of alcohol later in life (Grant, 1998; Chen et al., 2002; Jensen et al., 2003; Riala et al., 2004). In agreement with the literature, nicotine administration to animals can enhance subsequent self-administration of alcohol in them (Blomqvist et al., 1996; Smith et al., 1999; Le et al., 2003; Bito-Onon et al., 2011; Doyon et al., 2013a). However, there are also certain conflicting reports in which nicotine administration did not have any influence on alcohol consumption; rather nicotine administration reduced alcohol consumption (Dyr et al., 1999; Nadal and Samson, 1999; Sharpe and Samson, 2002).

There are various diverse molecular targets throughout the central nervous system (CNS) on which ethanol and nicotine act, but a common pharmacological action is also shared by these drugs (Dani and Bertrand, 2007; Dopico and Lovinger, 2009). It is put forward that common modulation of the brain stress hormone system and mesolimbic dopamine (DA) system leads to the interactions between ethanol and nicotine (Larsson and Engel, 2004; Funk et al., 2006; Doyon et al., 2013b). Substances for abuse (drugs) target the DA system and the development of addiction is due to the dysregulation of the DA system (Luscher and Malenka, 2011; Sulzer, 2011).

Increased susceptibility to drug and alcohol abuse can be associated with blunted transmission of DA (Volkow et al., 1996; Martinez et al., 2005; Ostroumov et al., 2015). By modifying the function of neural substrates that are targets of both alcohol and nicotine, nicotine can influence consumption of alcohol. Some examples of these substrates are stress hormone systems linked with glucocorticoids and corticotropin releasing factor (CRH), and the mesolimbic DA system (Larsson and Engel, 2004; Funk et al., 2006; Doyon et al., 2013b; Ostroumov et al., 2015). Ethanol and nicotine both have complex pharmacological actions and act on several targets present in the nervous system. According to different studies, reinforcing effects of ethanol and nicotine combination might arise from multiple mechanisms and different areas of the brain (Leao et al., 2015; Ostroumov et al., 2015).

## Alcohol and Cannabis

A 9-year survey was done between 2002 and 2010 by questionnaires about the use of alcohol, stimulants, and cannabis in 3,099 human immunodeficiency virus (HIV)-infected men and the study was termed as Veterans Aging Cohort Study (VACS) (Adams et al., 2018). In this study, the changes in the VACS index were analyzed by the above-mentioned substances. Alcohol and narcotic drugs influenced on progression of HIV disease by mechanisms like poor adherence to pharmacological therapy of HIV, increase in symptoms of depression, immune suppression, neurocognitive dysfunction, and respiratory infections (Arnsten et al., 2002; Kapadia et al., 2005; Hinkin et al., 2007; Sullivan et al., 2011; Langebeek et al., 2014; Kalichman et al., 2015; Adams et al., 2018). A fact that is important regarding the health of public is that drug and alcohol use is common among HIV-infected individuals (Chander et al., 2006; Mimiaga et al., 2013).

In general, cannabis use does not impact mortality in a negative manner, whereas there is a greater risk of mortality associated with stimulant use as compared to a lower risk of alcohol use among men infected with HIV in care (Adams et al., 2018). Association between stimulant use and mortality risk can help in its treatment in a targeted manner. Also the knowledge that frequent use of stimulant can lead to dangerous consequences can help patients to reduce or stop the use of stimulants. There is a greater impact of sociodemographic characteristics on mortality risk as compared to stimulants, alcohol, or cannabis use. Reduction of impact of racial differences and poverty by specific programs can be useful in improving the health of male veterans suffering from HIV/AIDS (Adams et al., 2018). Still, sufficient studies are required to draw conclusive remarks for effect of the cannabis use and alcohol consumption.

## Brief History of Alcohol Consumption

Although opinion differs as to when the humans first started to produce or became familiar with alcoholic beverages, their use dates back to ancient civilizations. Substantive historical and archaeological evidence implies the Stone Age [8000 Before the Common Era (BCE)] as the dawn of fermentation products (Guidot and Mehta, 2014). It was fermented mare's milk in ancient Siberia that appears to have been the first alcoholic drink. Its production today known as “Kumis” continues in some parts of Russia (Guidot and Mehta, 2014). The use of alcoholic beverages is reported in various religious ceremonies, social gatherings, or in day-to-day life.

Several excavation sites around the globe unearthed jars—dating it back to 7000–6600 BCE Northern China (McGovern, 2013), 5400–5000 BCE in Hajjin Firuz in Iran (Gately, 2008), 4000 BCE in ancient Egypt (Lucia, 1963), 2700 BCE in Babylonians (Hyams, 1965), 1000 BCE in Mexico (Gately, 2008), and 700 BCE in Greece (Hanson, 2013)—that were used for storing alcoholic beverages that were prepared form grapes, berries, rice, honey, wheat, and barley. With the commencement of the second and first centuries BCE, alcohol intoxication was no longer rare among the common people. India and China have very well-established and extensively documented traditional medicine systems [Ayurveda and traditional Chinese medicine (TCM)] counteracting the ill-effects of alcohol consumption.

### History of Alcohol Consumption in China

China has a rich legacy of fermentation products. Of the several recipes used for different products, one of the prominent being the beer recipe, that is in use over 5,000 years, made by fermenting ingredients such as tubers, Job's tears, barley, and broom millet (Wang et al., 2016). Some scholars have put forward the hypothesis that beer brewing by Shang tradition has its roots in the Neolithic Yangshao period (5000–2900 BCE), which dates back to the time of numerous agricultural settlement in the Yellow River Valley (Li, 1962; Huang, 2000; Wang et al., 2016).

Also, there is a similarity in terms of style in brewing vessels found in the Yangshao period like *jiandiping* (pointed-bottom vessel) amphorae and funnels, and those found in the modern ethnographic records and the historical period (Wang et al., 2016). But there is no direct confirmation of alcohol production from the Yangshao sites. There is a link between beer brewing and an increase in complexity in social structure marked by competitions among particular settlements, their hierarchical structure, and construction of large public buildings. Consumption of alcohol became common during feast days and rituals, with the beverages being financed by the elites, which was especially true of the late Yangshao period in the Wei River region (Liu, 2005), an area known as “the cradle of Chinese civilization” (Wang et al., 2016).

### History of Alcohol Consumption in India

Alcohol consumption in India was in practice since the ancient times. During the Vedic period (ca. 1500–700 BCE) alcoholic drinks were used in various religious festivals, consumed widely by warriors groups, and a few other sections of society (Achaya, 1991; Sharma et al., 2010). The use of this also continued during post-vedic era, during Islamic invasion, British rule, and significantly increasing in the present scenario (Sharma et al., 2010). During the Vedic period, alcohol consumption is marked by evidence gathered by excavation of chief ingredients from various archaeological sites, which implies alcohol was produced more than 4,000 years ago, i.e., it was contemporary with ancient civilizations of Mesopotamia, Egypt, and China.

Vedic literature refers to alcoholic beverages as *soma* and *sura* with the former being considered a sacred drink, and the latter—the drink of the common people. Alcohol consumption in the ancient days had certain restrictions: while Kshatriyas (warriors) were allowed to consume alcohols, Brahmins were completely forbidden to do so. In the post-Vedic period (700 BCE–1100 CE), the tradition of drinking alcohol continued and it was served on special occasions like moving into a new house or during weddings. Also, we find alcohol usage mentioned in the epic book of *Ramayana* and *Mahabharata* (Prakash, 1961; Singh and Lal, 1979; Boesche, 2002; Sharma et al., 2010). In the Mauryan period of Indian history (4^th^ century BCE), the production and sale of alcohol was under strict control and there were special houses set up for drinking. Some of these facts have been mentioned by *Kautilya* (prime minister of Chandra Gupta Maurya). *Kautilya* has also mentioned the names of various alcoholic preparations like “prasanna” and “medhaka” made from fermented wheat flour and rice, respectively (Achaya, 1991; Boesche, 2002; Sharma et al., 2010).

During this period some sections of society like the *Tantric* sect incorporated the use of alcohol as an essential part of their religious ceremonies. They made *madya* (wine) an essential component of their *ganachakra* (tantric assembly). Two main medical practitioners of the post-Vedic India were *Charaka* and *Sushruta*. Charaka wrote that alcohol in right amounts at a right time and with enough food is beneficial. He posited that moderate drinking leads to preservation of intelligence, provides nourishment, digestion and pleasure, while *Sushruta* wrote about the use and abuse of alcohol.

Consumption of alcohol during the Islamic rule comes out with stick to prohibition, as liquor is forbidden in Islam; but still wine was used on a regular basis in royal or princely courts. At the start of the British and European colonial rule in India opium and cannabis were more popular, but slowly under the patronage of Europeans alcohol consumption began to thrive in India. A new brand of beer was developed by George Hodgson (London) for India, which was light in nature and had a bitter taste so later beer in India began to be known as “Indian Pale Ale.” Also Edward Dyer from England established a brewery in Kasauli in the Himalayan region and it was called Dyer's Lion Beer and was credited with being the first commercially produced beer in Asia. Moreover, it found popularity among British troops. At the end of the 19^th^ century the Indian movement for independence grew stronger and leaders like Balgangadhar Tilak (during the first decade of 20^th^ century) urged the people to start boycotting British government licensed liquor shops. Also, women like Kasturbai, also known as Kasturba Gandhi (wife of Mahatma Gandhi) led strong movements against liquor sale and consumption. Alcohol consumption at this moment of time was considered a bad habit associated with the British and was highly condemned by women organizations, nationalists, and others. Alcohol consumption was spread by Indian soldiers and office clerks who served for the British and embraced the western culture of consuming alcohol (Wolpert, 1997; Saxena, 1999; Parkar et al., 2001; Benegal, 2005; Pryor, 2009; Sharma et al., 2010). An independent India imposed various laws and regulations on the production and sale of liquor, differing from state to state. Still, the consumption of alcohol continued to increase, and in between 2010 and 2017 a net increase of 38% was recorded from 4.3 to 5.9 L per adult per year. Apart from this, there are several states such as Andhra Pradesh, Gujarat, and Bihar where there are complete prohibition of alcohol.

In today’s scenario consumption of lighter drinks like wine and beer is increasing at a remarkable rate with time. Drinking at a young age and social drinking is gaining popularity day by day and this is reflective of the changing Indian society. Many restaurants, bars, and social places have come up in India to meet social and urban drinking demands. Though people in India are now beginning to follow the concept of moderate and safe drinking, still a large percentage of people in India consume alcohol in a manner that is hazardous to them and the society. Education and proper counselling regarding alcohol consumption and the attendant dangers will have a bigger role to play in coming years to overcome this changing scenario in changing Indian society (Chandra et al., 2003; Singh and Bloom, 2004; Verma et al., 2004; Benegal, 2005; Sivaram et al., 2008; Sharma et al., 2010).

## Alcohol Abuse and Social Implications

The alcohol use disorder (AUD) afflicts 20–30% of men and 10–15% of women throughout the world according to the data compiled by the American Psychiatric Association (APA, 2013; Grant et al., 2015). A recent report of WHO estimated that in 2016, over half (3.1 billion people or 57%) of the global population over 15 years of age had abstained from drinking alcohol in the last 12 months and around 2.3 billion people are currently drinkers (WHO, 2019). Moreover, substantive figure, i.e., half of the populations of Americans, Europeans, and Western Pacific countries were indulged in alcohol consumption (WHO, 2019). According to Global Burden of Diseases, Injuries, and Risk Factor Study (GBD, 2016), data compiled from 1990 to 2016 for 195 countries and territories demonstrated that alcohol was the major contributor leading to death, disability, and bad health. In 2016 alone, the cause of death and disability due to alcohol consumption stands at the seventh leading risk factor, accounting to about 2.2% of female deaths and 6.8% of male deaths. But when the data was sub-categorized for a special category of age group between 15 and 49 years, the scenario transformed from the seventh leading risk factor to the leading cause of deaths. The attributable death count stands at 3.8% for females and 12.2% for males.

Similarly, Borges and co-worker reported dose-response estimates for the odds ratio (OR) and population attributable risk of acute alcohol consumption and road traffic injury (RTI) (Borges et al., 2017). In this study the data was obtained and analyzed from 1,119 RTI patients who reached 16 emergency departments in countries like Trinidad and Tobago, Dominican Republic, Brazil, Guatemala, Costa Rica, Guyana, Nicaragua, Panama, Mexico, and Argentina. The results of the study highlights that 1 in every 6 RTI patients in emergency department agreed to have alcohol intake 6 h prior to injury. This figure was five times higher when compared to not drinkers. Thus, decreasing the intake of alcohol to low to moderate levels (≤4 drinks) had significant impact on population burden and risk.

Applying local and global measures immediately in areas like Latin America and the Caribbean to decrease consumption of alcohol can reduce usage of alcohol among pedestrians, drivers, and passengers involved in RTI (WHO, 2010; Borges et al., 2017).

Due to low availability of methods to prevent alcohol consumption and treatment of alcohol-caused disorders in low- and middle-income countries, an e-health portal was launched by the World Health Organization (WHO) on December 6, 2012 relating to alcohol and health in a web-based self-help program. In such countries, the introduction of an effective e-health program can lead to a positive impact on people's health, as they provide self-help to people regarding alcohol consumption and the health complications related to it (Dedert et al., 2015).

Cognitive–behavioral therapy and self-help health programs have had significant effect among the programs targeting alcohol abuse in countries with high economic status (Riper et al., 2011; Riper et al., 2014; Sundstrom et al., 2017). Web-based programs are easily accessible for individuals who are at a high risk for developing disorders based on alcohol consumption and are supposed to prevent further health complications (Riper et al., 2011). Also some drinkers (referred to as hidden drinkers) who usually do not contact any health professionals for one reason or another can profit from these web-based programs and this is of great importance to the public health (Schaub et al., 2016; Schaub et al., 2018).

## Ethnopharmacological Applications Targeting Alcohol Abuse

This limited efficacy and associated adverse effects have urged us to deepen our understanding of the complementary approaches used in traditional and folk medicine. Notably, recent experimental evidence has proved the effectiveness of some herbal remedies (Carai et al., 2000; Xu et al., 2005; Abenavoli et al., 2009) with few possible side effects, and natural products in general are an established source of pharmacologically active molecules (Atanasov et al., 2015; Yeung et al., 2018).

The use of traditional products/formulations aims to target at: (a) reducing the desire to drink; (b) impeding gastrointestinal absorption of alcohol; and (c) expediting the process of alcohol and its metabolites clearance rate form the body (Xu et al., 2005).

XJL [Natural Pharmacia International [NPI] preparation #28 (NPI-028)] is an herbal medicine developed in China and has been used for decades to decrease the intoxicating effects of alcohol. Extracts of *Pueraria montana* var. *lobata* (Willd.) Maesen & S.M.Almeida ex Sanjappa & Predeep (syn. *Pueraria lobata* (Lour.) Merr. (kudzu) and *Citrus × aurantium* L. (syn. *Citrus reticulata*) are among the many plant extracts that have been used in preparing XJL. The exact mechanism of action of kudzu is unknown but studies carried out on isoflavones like daidzein, daidzin, and puerarin (phytochemicals found in kudzu extract) have concluded that these phytochemicals decrease the consumption of alcohol by alterations in monoamine oxidase (MAO)-acetaldehyde pathways or mitochondrial ALDH2 pathways (Keung, 2003; Lukas et al., 2013).

*Chunggan* extract (CGX) is a commercially marketed herbal medicine of 13 herbs, which finds its utility as a potent “liver cleaning” agent (Choi et al., 2006; Kim et al., 2014). Kim et al. (2014) observed pharmacological properties of CGX with the main focus on molecules related to alcohol metabolism and pro-fibrogenic cytokines. They also saw the mechanism in rat-derived HSC cell line (using HSC-T6 cells) (Kim et al., 2014).

**Figure 1:**
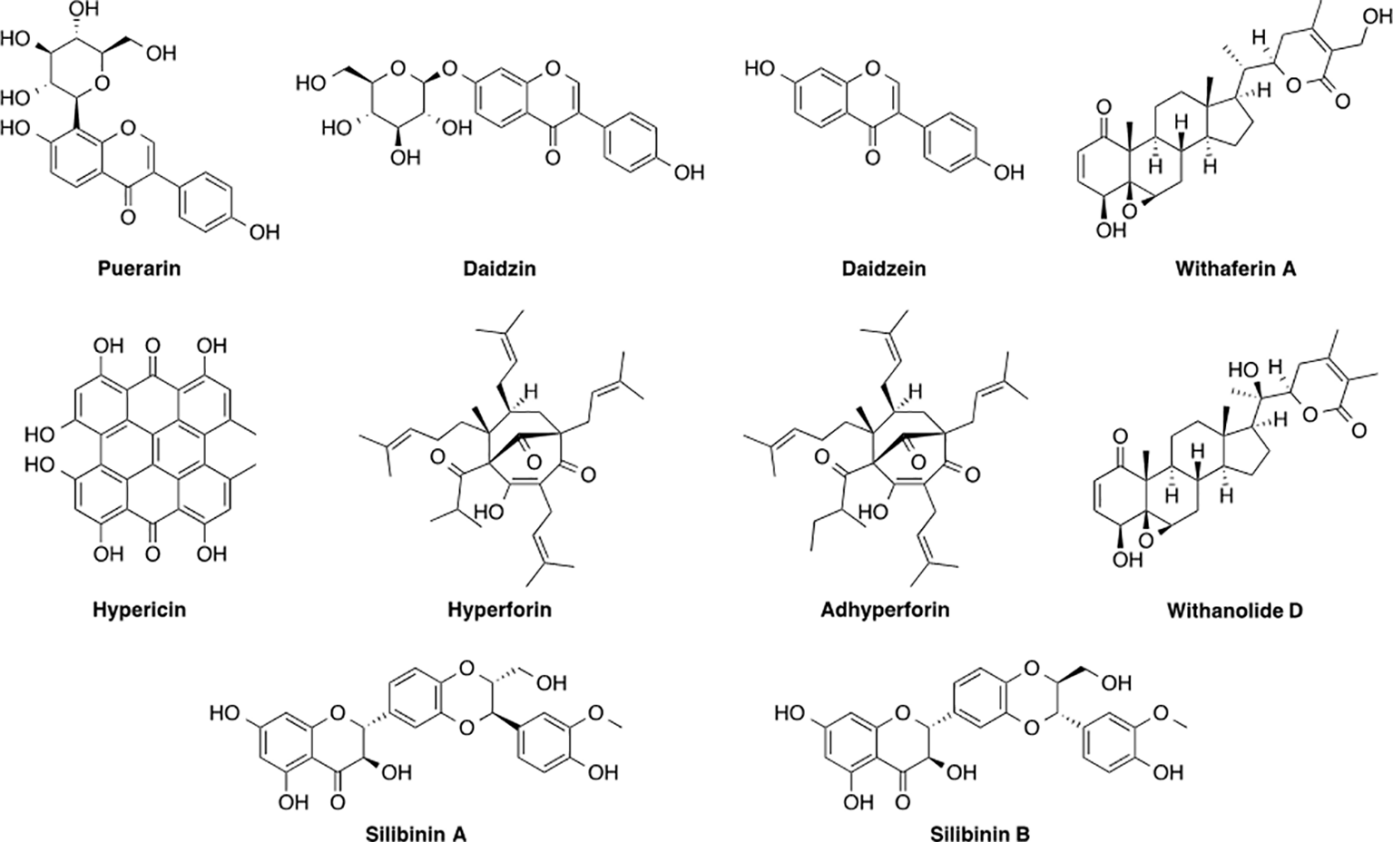
Chemical structures of some important natural products.

Recent experimental development to the application of herbal and traditional medicines has led to the isolation and characterization of pure and active compounds such as daidzin, daidzein, and puerarin from *Pueraria montana* var. *lobata* (Willd.) Maesen & S.M.Almeida ex Sanjappa & Predeep, iboganine from *Tabernanthe iboga* Baill., tanshinones I and II, cryptotanshinone, and miltirone from *Salvia miltiorrhiza* Bunge, hyperforin from *Hypericum perforatum* L., ginsenosides from *Panax ginseng* C.A.Mey., and withanolide D and withaferin A from *Withania somnifera* (L.) Dunal etc., that are some of the widely studied and well-known species suppressing alcohol intake in experimental animals (Sweetnam et al., 1995; Butterweck et al., 1997; Rezvani et al., 2002; Abenavoli et al., 2009; Zhu et al., 2017). These substances are known to put forth their effects by influencing several of the neurological systems, thereby suppressing drinking behavior. In this section, **Table 1** provides a brief summary on various plant species and natural products derived from them and their proposed mechanisms of action in the context of alcohol intake. The chemical structures of some of the important discussed natural products are presented in **Figure 1**.

**Table 1:** Plants used for prevention and treatment of alcohol abuse in different folk medicine practices.

Botanical name	Main phytochemical structure	Possible mechanism
*Pueraria montana* var. *lobata* (Willd.) Maesen & S.M.Almeida ex Sanjappa & Predeep (syn. *Pueraria lobata* (Lour.) Merr.)	Isoflavones derivatives (daidzin, puerarin)	1. Reversible inhibition of mitochondrial ALDH-2 and increase of 5-hydroxyindole 3- acetaldehyde (5-HIAL) (Keung and Vallee, 1998a; Keung and Vallee, 1993b; Lu et al., 2009). 2. Alteration of BDZ receptors positioned on GABA-chloride channel complex (Shen et al., 1996; Keung and Vallee, 1998). 3. Alcohol-induced inhibition and disruption of hippocampus function leading to the suppression of c-fos protein (FOS) expression (Jang et al., 2003; Rezvani et al., 2003).
*Salvia miltiorrhiza* Bunge	Phenanthrenequinones compounds including cryptotanshinone, tanshinones I, II, and miltirone	1. Militirone, low-affinity ligand for central GABA_A_-BDZ–binding site, thus acting as a partial agonist and implying an anxiolytic effect (Lee et al., 1991). 2. Miltirone partly inhibits upsurge in mRNA levels of the α 4 subunit of GABA that was persuaded through ethanol withdrawal in cultured hippocampal neurons (Mostallino et al., 2004; Zhu et al., 2017). 3. Tanshinone IIA improves alcoholic liver disease by decreasing lipopolysaccharide and Kupffer cell sensitization induced by alcohol (Yin et al., 2008). 4. Cryptotanshinone inhibits ALD through hindering fatty acid synthesis and hepatic cell death (Yin et al., 2009).
*Hypericum perforatum* L.	Phloroglucinol derivatives (adhyperforin, hyperforin), and anthraquinone derivatives (hypericin, pseudohypericin)	1. Inhibits the uptake of serotonin and noradrenaline (aminergic transmitters) in the synaptic nerve endings (Butterweck et al., 1997; Kumar et al., 2006). 2. Increase in level of serotonin, dopamine, norepinephrine or through stimulation of opioid and sigma receptors in the CNS (Müller et al., 1997; Panocka et al., 2000).
*Panax ginseng* C.A.Mey.	Ginsenosides	1. Increase of metabolism of alcohol and decreased blood alcohol levels (BALs) by enhancing ADH activity and plasma clearance (Lee et al., 1993). 2. Incite the microsomal ethanol-oxidizing system and ADH action and thereafter fasten the removal of acetaldehyde while shunting the excessive hydrogen into lipid biosynthesis (Abenavoli et al., 2009).
*Tabernanthe iboga* Baill.	Ibogaine	1. Suppressive effect on alcohol intake by regulating several neural pathways particularly dopaminergic and serotonergic systems (Deecher et al., 1992; Sweetnam et al., 1995; Overstreet et al., 2003). 2. Interacts with k-opiate receptor and inhibits k- receptor mediated dopamine release in rats (Deecher et al., 1992; Reid et al., 1994).
*Withania somnifera* (L.) Dunal	Withanolide D and withaferin A	Blocks GABA receptors binding and up-surges chloride influx in absence of GABA (Gupta and Rana, 2008; Lu et al., 2009; Ruiu et al., 2013).
*Macropiper methysticum* (G.Forst.) Miq. (syn. *Piper methysticum* G.Forst.)	*Kava* lactones	1. Binding to multiple locations in the brain and interaction with different neurotransmitters and significant inhibition of the uptake of noradrenaline, but not serotonin (Sällström Baum et al., 1998). 2. Also affects the concentration of dopamine and its metabolites that is/are in turn associated with altered behavioral response in rats (Sällström Baum et al., 1998).
*Thunbergia laurifolia* Lindl.	Iridoid glucosides of 8-epi-grandifloric acid and 3'-*O*-β-glucopyranosyl stilbericoside	1. Increase of blood flow signals in amygdala, nucleus accumbens, frontal cortex, and caudate putamen (areas in the brain linked with addictive drug pathways) (Thongsaard et al., 2005). 2. Shares similarity with amphetamine in increasing potassium-triggered dopamine release from rat striatal slices, suggesting the potential efficacy for addictive drugs is dopamine-dependent (Thongsaard and Marsden, 2002).
*Banisteriopsis caapi* (Spruce ex Griseb.) Morton	Beta-carbolines, such as harmine, harmaline, and tetrahydroharmine (THH)	1. Harmine and harmaline showed substantial inhibitory (*in vitro*) activity against MAO-A and -B in human brain and stimulate dopamine release (Samoylenko et al., 2010). 2. THH can also inhibit serotonin reuptake (Samoylenko et al., 2010).
*Corydalis yanhusuo* (Y.H.Chou &Chun C.Hsu) W.T.Wang ex Z.Y.Su & C.Y.Wu	Lev-tetrahydropalmatine (L-THP)	1. *L*-THP inhibits oxycodone-induced hyperactivity (Liu et al., 2005). 2. Anti-addictive properties may be due to dopamine transmission antagonism. 3. Inhibits dopamine receptors D1 and D2 and acts upon the nigra-striatal neuronal pathways and inhibits pre- and post-synaptic receptors (Marcenac et al., 1986; Jin, 1987). 4. Prevents L-type Ca^2+^ channels inhibition; here it is notable that L-type Ca^2+^ channel inhibition is vital for the development of drug tolerance, sensitization, and dependence (Jin, 1987).
*Lophophora williamsii* (Lem. ex SalmDyck) J.M. Coult.	Mescaline (3,4,5-trimethoxy-β-phenylethylamine)	The mescaline molecule is structurally similar to serotonin and acts on the serotonin (5-HT_2A_) receptor. 5-HT_2A_ receptors activation increases cortical glutamate levels apparently through a pre-synaptic receptor-mediated release from thalamic afferents (Nichols, 2004; Gibbons and Arunotayanun, 2013).
*Hovenia dulcis* Thunb.	Ampelopsin, hovenitins I, II, & III, laricetrin, myricetin, and gallocatechin	1. Decrease of gastrointestinal absorption of alcohol and reducing of blood alcohol concentration (Xu et al., 2005). 2. Effective in enhancing ALDH activity than ADH activity, blocks lipid peroxidation, and eradicates unwarranted free radicals produced by alcohol (Yoshikawa et al., 1996b; Hase and Basnet, 1997; Xu et al., 2004).
*Oenothera biennis* L.	γ-linolenic acid (GLA)	1. Excess alcohol consumption hinders the metabolism of GLA, which is a precursor of prostaglandins. As a result, prostaglandins E1 (PGE1) levels are reduced in alcohol addicts, often leading to depressive states that increase patients' inclination to drink. The need to drink is thus indirectly lowered by a reduction in the depression symptoms (Tomczyk et al., 2012). 2. Protects liver and kidney damage caused by alcohol intake by counteracting the enzyme inhibition (Glen et al., 1987; Abenavoli et al., 2009; Tomczyk et al., 2012)

## Methodology

We collected and documented scattered information on counteracting of alcohol abuse through medicinal plants. The synonyms of the various species were cross-checked with the plant databases (https://mpns.science.kew.org). Afterwards, the available articles on respective species were retrieved using popular search engines and various databases, for instance, Scifinder, Science Direct, PubMed, Scopus, Mendeley, JOAP, Microsoft Academic, and Google Scholar. The keywords used were alcohol dependence, addiction, complimentary medicines, phytotherapy, ethnopharmacology, and ethnobotany, alcohol dehydrogenase enzyme, dopamine, gamma-amino butyric acid, etc. The data was congregated through the Boolean information retrieval method using plant name along with “AND” operator (Pohl et al., 2010; Tewari et al., 2017a) followed by alcohol dependence and addiction. No prerequisite limitations on publications, i.e., language, year, and publication type (original contribution, review article or key editorial note), were taken into consideration. An outline of the various plants species used for prevention and management against alcohol abuse is presented in **Table 1**.

## Prominent Medicinal Plants/Extracts for Management of Alcohol Dependence and Abuse

### *Pueraria montana* var. *lobata* (Willd.) Maesen & S.M.Almeida ex Sanjappa & Predeep

*P. montana* var. *lobata* (Willd.) Maesen & S.M.Almeida ex Sanjappa & Predeep (syn. *Pueraria lobata* (Lour.) Merr.) (Fabaceae), commonly known as kudzu, is a perennial climbing plant distributed throughout Asia (including Japan, Korea, China), as well as in some regions of North and South America. Kudzu is a noxious weed in the United States and it has been used for several centuries as *Puerariae radix* in traditional Chinese medicine. The plant has been known since the Pharmacopeia of Shen Nong (about 200 BC) in China and used as antidiarrheal, antiemetic, diaphoretic, and antipyretic agent (Zhu et al., 2017). Apart from this, the species finds its utility in treatment of fevers, muscle aches, gastrointestinal disorders, skin problems, allergies, high blood pressure, and chronic alcoholism (Lee et al., 1999; Abascal and Yarnell, 2007).

Chinese Pharmacopeia of 600 AD describes its application in treatment of alcohol intoxication (Sun Simiao, about 600 AD) and as an anti-dipsotropic agent (Li Dongyuan, about 1200 AD). Crude extract of the species is a significant source of physiologically valuable isoflavones including puerarin, daidzin, and daidzein (Ohshima et al., 1988), compounds notably reported to be useful in suppressing free-choice ethanol intake in Golden Syrian hamsters (Keung and Vallee, 1993a), Wistar rats (Heyman et al., 1996), Fawn Hooded rats (Overstreet et al., 1996), and alcohol-preferring (P) rats (Lin et al., 1996; Benlhabib et al., 2004a).

The probable neurological pathway acting against ethanol intake is said to be mainly due to daidzin, a selective and reversible mitochondrial ALDH-2 inhibitor. It is crucial for acetaldehyde oxidation, which is a resultant of ethanol metabolism by suppression of reactive intermediates 5-hydroxyindole-3-acetic acid (5-HIAAA) and 3,4-dihydroxyphenylacetic acid (DOPAL) formation from serotonin and dopamine (Keung and Vallee, 1998), as a result of which there is an increase in levels of 5-hydroxyindole 3-acetaldehyde (5-HIAL) and DOPAL. Thus, daidzin appears to stifle alcohol intake through aggregating 5-HIAL and inhibiting ALDH-2 (Keung, 2003).

Shen et al. (1996) studied the pharmacological effects of daidzin and puerarin on ethanol via GABA/BDZ-chloride channel complex. BDZ tranquilizers are known to modulate the efficiency of inhibitory neurotransmitter GABA at the GABA/BDZ-chloride channel complex in the brain (Harris, 1990; Lin et al., 1993).

The *in vitro* use of daidzin and puerarin showed a mixed competitive and non-competitive inhibition of [^3^H] flunitrazepam binding to cortical, cerebellar, and hippocampal membranes in the brain. Alcohol is known to modulate the brain's mechanism to inhibit neurotransmitter GABA by chloride channel complex. Hence, kudzu extract can be used as an agent for treating alcohol dependence by virtue of its actions on BDZ receptors and role in modification in monoamine metabolism.

In a recent study, Penetar and colleagues administered kudzu extract after an episode of acute drinking, in order to check the rate/level of ethanol concentration in blood (Penetar et al., 2011). Similarly, Shen and co-workers, using rats as the experimental animal, demonstrated that dihydromyricetin (DHM) [(2*R*,3*R*)-3,5,7-trihydroxy-2-(3,4,5-trihydroxyphenyl)-2,3-dihydrochromen-4-one], a flavonoid component of kudzu extract, is effective against acute ethanol intoxication, thereby increasing alcohol withdrawal symptoms possibly by GABA_A_ receptor-mediated mechanism. The increased hippocampal expression of GABA_A_ receptor and interaction of DHM with the BDZ site on the GABA_A_ receptor were held responsible for the above-mentioned actions of DHM (Shen et al., 2012).

The extract of kudzu has an excellent ability to change the effects of alcohol or reduce alcohol consumption in animals used in laboratories (Heyman et al., 1996; Keung and Vallee, 1993a; Overstreet et al., 1996; Keung, 2003). The extract can decrease the alcohol intake by up to 50% and the effect develops within only 1 to 2 days. It is an accepted fact that the isoflavones of kudzu extract have an ability to decrease alcohol consumption in a variety of mammalian species, regardless of the mechanism of action (Lukas et al., 2013).

There are several advantages of kudzu extract treatment over other medicines; for instance, it has minimal adverse effects when compared to its synthetic counterparts (Lukas et al., 2013).

### *Salvia miltiorrhiza* Bunge

*Salvia miltiorrhiza* Bunge (Lamiaceae), commonly named as Danshen or Red Sage root, is a perennial medicinal herb that is extensively used across Asia. The name “salvia” is derived from a Latin word “salvere” meaning “to heal,” which is in line with the folkloric belief regarding it as a plant with “magical” healing properties (Kasimu et al., 1998). The species is highly valued for its root and is classified as ‘‘super grade herb (herbs without observable toxicity)'' in Shennong's Herbal Classic of Materia Medica (Shennong Bencao Jing about 221 BC to 220 AD) and has been utilized for over 2000 (Wang et al., 2017). It is listed in the Chinese Pharmacopoeia for treatment of menostasis, menstrual disorder, insomnia, menorrhalgia, and blood circulation diseases (China Pharmacopoeia Committee 2005). Recent investigation advocated its utility in different substance abuse, such as abuse of tobacco, alcohol, and various other drugs (Mah et al., 1998). The extract of the species is reported to be useful in the treatment of liver diseases, acquired immunodeficiency syndrome (AIDS), diabetic nephropathy, etc. (Abd-Elazem et al., 2002; Peng et al., 2018).

The major chemical constituents of *S. miltiorrhiza* roots are diterpene pigments, of orange–red color, with a phenanthrenequinone structure, such as tanshinones I, II, cryptotanshinone, and miltirone (Serra et al., 2003). Other biologically active constituents include danshensu, protocatechualdehyde, rosmarinic acid, and salvianolic acid A (Luo et al., 2015). Over 30 tashinone compounds are isolated from *S. miltiorrhiza* and that have antioxidant, antitumor, antiplatelet, and antiviral properties (Zhang and Wang, 2006). Pharmacological properties of some of these molecules have been well explored. Compounds such as tanshinones I and II, miltirone, and cryptotanshinone are known to play a key role in reducing alcohol intake in model animals (Carai et al., 2000; Serra et al., 2003), thus promoting antirelapse in alcohol-preferring rats (Colombo et al., 2006).

Lee et al. (1991) reported militirone as a low-affinity ligand for central GABA_A_-BDZ–binding site responsible for many behavioral consequences of alcohol, and hence acting as a partial agonist and having an anxiolytic effect (Lee et al., 1991). Similarly, IDN 5082 (standardized extract of Danshen) inhibited discriminative stimulus effects of ethanol in Sardinian alcohol-preferring (sP) rats (trained to distinguish ethanol from water) (Colombo et al., 1999) and substantially suppressing ethanol acquisition (Serra et al., 2003), an indication of its antirelapse properties (Serra et al., 2003). Likewise, Koo et al. (2004) reported that Danshen crude water extracts stimulated potassium-dependent release of dopamine from striatal slices of rats comparable to amphetamine (a stimulant drug) suggesting a probable mechanism of action that might be useful for substance abuse pharmacotherapy. Similarly, tanshinone II, an active constituent of the root extract, is reported to ameliorate ALD activity by reducing lipopolysaccharide and ethanol-induced Kupffer cell sensitization (Yin et al., 2008), while crypto-tanshinone is reported to inhibit ALD by blocking hepatic cell death and fatty acid synthesis (Yin et al., 2009). Likewise, salvianolic acid B relieved acute ethanol-induced hepatocyte apoptosis through sirtuin 1-mediated deacetylation of the p53 transcription factor pathway (Li et al., 2014).

### *Hypericum perforatum* L.

*Hypericum perforatum* L. (Clusiaceae) commonly named as St. John's wort (SJW) is an herbaceous perennial plant, with extensive creeping rhizome. The word *hypericum* derives from Greek “hyper” (over) and “eikon” (image or apparition), which reflects the herb's use against evil spirits. The species is native to Europe (Klemow et al., 2011), and widely distributed across the temperate areas of North Africa, Asia, Australia, and America (Gleason and Cronquist, 1963). Due to putative medicinal property it was recommended against various disorders from the first century onwards by various Greek physicians (such as Dioscorides, Galen, Hippocrates, and Pliny). Ever since then it has endured as a popular remedy for intestinal worms, anxiety, cuts, burns, depression, snakebites, and menstrual disorders (Castleman, 2001; Redvers et al., 2001). The species is also known to possess hypotensive, antibacterial, spasmolytic, stimulating (Chopra and Nayar, 1956), diuretic, (Solujic et al., 1997), analgesic, anti-inflammatory (Bukhari et al., 2004), anticancer, antitumor, antioxidant, antischizophrenic, anticonvulsant, and antidiabetic properties (Birt et al., 2009; Caraci et al., 2011; Can and Ozkay, 2012).

The species has been widely studied for its chemical composition and pharmacological properties. As a result, many representatives of bioactive compound groups have been isolated and described, including hypericin and pseudohypericin (naphtodianthrones), hyperforin, adhyperforin (phloroglucinol derivatives), several flavonol glycosides, proanthocyanidins, phenylpropanes, biflavones, xanthones, tannins, and some amino acids (Bombardelli and Morazzoni, 1995; Barnes et al., 2001).

SJW’s use is popular as an herbal remedy due to its efficacy against many diseases, especially depression and alcohol dependence. Recent experimental and clinical studies have identified hyperforin as an active biological compound with antidepressant qualities (Abenavoli et al., 2009), also being a good signature agent for reducing alcohol intake and desire to drink (Perfumi et al., 2001).

In-depth study on alcoholism and depression led to the identification of a common neurochemical substrate for both disorders (Markou et al., 1998), whereby coexistence of two or more neurochemical events was traced amid high alcohol intake and a depression-like condition in experimental alcohol-preferring rats. Ballenger et al. (1979) thought that depression and alcoholism might result from low serotonin levels that are augmented transiently by alcohol intake (Ballenger et al., 1979). Serotonin-targeting compounds reduced pathological drinking in experimental animals (Murphy et al., 1988; Rezvani et al., 1991; Rezvani and Grady, 1994). Notably, other preclinical studies have suggested that SJW has a role in reducing voluntary intake of alcohol. These works base their assertion on the tests carried out on selectively bred alcohol-preferring rats (De Vry et al., 1999; Panocka et al., 2000), Fawn-Hooded (FH) rats (Overstreet et al., 1992; Rezvani et al., 2002), high alcohol-drinking (Had) rats, Marchigian Sardinian (msP), and sP rats (Ciccocioppo et al., 1999).

Likewise, from other clinical and experimental studies, hyperforin, a lipophilic constituent of SJW, was found to inhibit aminergic transmitter's uptake of serotonin and noradrenaline into synaptic nerve endings (Kumar et al., 2006). It was also known to increase the level of norepinephrine, dopamine, serotonin, and GABA in the brain.

Other than this, the probable anticraving and antidepressant effects of SJW have been suggested to be as a result of increase in the levels of serotonin, dopamine, norepinephrine, or by sigma and opioid receptors stimulation in the CNS (Butterweck et al., 1997; Müller et al., 1997).

### *Panax ginseng* C.A. Mey

*Panax ginseng* C.A. Mey (Araliaceae) is a well-known perennial herb, documented as ginseng in the traditional Chinese system of medicine. Its name is derived from the Greek words “pan” (all) and “axos” (cure) meaning possess of an inherent property to “cure all diseases.” The species is widely distributed in northeastern regions of the Korean peninsula (Park et al., 2005). For 5000 years the species has been used in certain parts of the world especially in Korea, China, and Japan. It is both a nourishing and tonifying agent and a potent therapeutic agent for many diseases like liver diseases, immune diseases, cancer, depression, fatigue, diabetes, internal degeneration, tumors, inflammation, nausea, dyspepsia, vomiting, nervousness, pulmonary problems, stress, and ulcers (Lee et al., 2005).

The pharmacological effects of ginseng are due to various bioactive molecules like ginsenosides, fatty acids, polysaccharides, peptides, peptidoglycans, phytosterols, triterpene saponins, and phenolic compounds. It is also known to contain essential oils, i.e., polyacetylenes and sesquiterpenes (Kim et al., 2017). Ginsenosides represent the unique and major pharmacological active constituents of ginseng that are said to be present as triterpene glycosides (Rastogi et al., 2015). Over 100 ginsenosides have been isolated from *Panax*, out of which 40 are found in *P. ginseng* alone (Christensen, 2009), mainly Rb1, Rb2, Rc, Rd, Rg1, Rg2, Rh1, and Re (Attele et al., 1999). Most pharmacological studies of ginsenosides were done for their immunostimulatory, anticancer, anti-inflammatory, antioxidative, prevention of opioid, and psychostimulant abuse and dependence (Tokuyama and Takahashi, 2001). However, little attention has been paid to alcohol intoxication.

Joo et al. (1982) proposed that ginseng saponins increased alcohol metabolism and lowered BALs by increasing ADH activity and plasma clearance (Joo et al., 1982). A few authors have reported on ginseng extract decreasing alcohol consumption, which was later confirmed by Lee et al. (1987). Clinical studies on volunteers demonstrated that in 10 out of 14 cases, ginseng extract accelerated alcohol clearance by 31–51%. Moreover, a recent study demonstrated that administration of red ginseng extract to alcohol-intoxicated rats altered alcohol absorption from the gastrointestinal tract (Carai et al., 2000) and prevented memory failure and excitation (Bao and Saito, 1984). It is also known to stimulate the microsomal ethanol-oxidizing system and the ADH enzyme action as a result of which there is a faster oxidation and removal of acetaldehyde with rapid shunting of excess hydrogen into lipid biosynthesis (Kwak and Joo, 1980). Thorough investigations are still needed concerning the value of ginseng in the treatment of alcoholism and associated problems, e.g., memory loss and nervous reactions.

### *Tabernanthe iboga* Baill

*Tabernanthe iboga* Baill (Apocynaceae), commonly named as Iboga, is a perennial rainforest shrub native to Western Central Africa. Its principal psychoactive compound ibogaine is making up 80% of the psychoactive compounds, and it represents indole alkaloid, which can be isolated from the roots of the plant. Some of the other compounds include ibogaline, which constitutes about 15%, ibogamine—up to 5% and—to a lesser extent—tabernanthine and vocangine (Kontrimaviciute et al., 2006; Maciulaitis et al., 2008).

Ibogaine, a psychoactive compound used for preclinical and anecdotal studies, has proved its prominent role in drug addiction therapy. Scrapings of iboga root bark with potent hallucinogenic and therapeutic properties have been used for centuries in various medicinal formulations, i.e., in small doses to combat hunger, fatigue, sleep, and thirst; in high doses it was used for spiritual experiences (Alper et al., 2008). Boiled leaves are applied in the treatment of toothache, latex—in the treatment of anthelmintic turmoil, and the roots as anesthetic and febrifuge agents (Pope, 1969). In the early 1960s, the psychotherapeutic effects of ibogaine were studied by a Chilean psychiatrist, Dr. Claudio Naranjo. He observed that ibogaine administration led to an active period of visualizing of past events often described as a “waking dream state” (Gallo et al., 2009). The exact mechanism by which this psycho-pharmacological drug affects the brain is poorly understood. Iboga alkaloids [i.e., ibogaine, noribogaine and 18-methoxycoronaridine (18-MC)] are reported to have multiple and complex mechanisms of action within the CNS (Alper et al., 2008). Ibogaine at low micromolar concentrations is reported to possess a binding affinity for several receptors present within the CNS, including glutamate, kappa, mu-opioid, and sigma_2_ receptors, *N*-methyl-D-aspartate (NMDA), sodium channels, and the serotonin reuptake transporter (Brown, 2013; Gallo et al., 2009; Mash et al., 2018).

Ibogaine has been effective in the treatment of different drugs abuse, including of morphine, cocaine, heroin, alcohol, and nicotine (Overstreet et al., 2003; Rezvani et al., 2003; Abenavoli et al., 2009). Ibogaine administration is known to cause a substantial reduction in drug withdrawal symptoms, a marked drop in the desire to use drugs; however, it can only be regarded as a simple initial element in the complete rehabilitation strategy. The preclinical studies support the use of the plant, whereby iboga alkaloids induced a significant reduction of opioid withdrawal signs in rats (Dzoljic et al., 1988; Maisonneuve et al., 1991; Parker et al., 2002; Panchal et al., 2005), in mice (Frances et al., 1992; Popik et al., 1995; Layer et al., 1996), and in primate (Leal et al., 2003). Iboga alkaloids are reported to decrease the self-administration of morphine (Pace et al., 2004), cocaine (Glick et al., 1994), amphetamine (Maisonneuve and Glick, 1992), methamphetamine (Glick et al., 2000; Pace et al., 2004), alcohol (Rezvani et al., 1995b; Rezvani et al., 1997), and nicotine (Glick and Maisonneuve, 1998; Glick et al., 2000).

Ibogaine is also said to be effective in treating alcohol dependence and abuse and was found to expressively reduce volitional alcohol consumption desire in alcohol-preferring FH, P, and AA rats. The anticraving effects of ibogaine are thought to be due to its ability to interact with the CNS and its ability to stimulate the dopamine and serotonin systems (Glick et al., 1991). Other than this, an analogue of ibogaine, i.e., 18-methoxycoronaridine (18-MC), displays the anticraving property in the same fashion as ibogaine by regulating dopamine and serotonin systems (Rezvani et al., 2003). In order to trace out the possible mode of actions or events happenings inside the brains, ibogaine and its analogue were tested. On systematic administration of iboga in alcohol-fed rats, the results showed that it significantly altered the level of dopamine and its metabolites within *nucleus accumbens*, striatum, and prefrontal cortex within the rat's brain (Sloviter et al., 1980; Maisonneuve et al., 1991), thus highlighting the anticraving property possessed. Similarly, on systemic administration of 18-MC it also resulted into decrease in extracellular levels of dopamine in *nucleus accumbens* of rats brain, thereby intimating its probable role in suppressing alcohol intake as off ibogaine. Other than this, the other possible mode of action of analogue 18-MC against alcohol intake includes its ability to associate and regulate the functional entity of opioids receptor, in the same fashion as ibogaine, which in turn interacts with k-opiate receptor (Deecher et al., 1992) and inhibits dopamine release (Reid et al., 1994). Thus, a possible suppressant effect on altering the endogenous opioid system is believed to counteract alcohol intake.

### *Withania somnifera* (L.) Dunal

*Withania somnifera* (L.) Dunal (Solanaceae) is commonly known as “Ashwagandha” or “Indian winter cherry.” It is regarded as a “Medhya rasayan” (Nootropic herb) in classical Ayurvedic system (Bhattacharya and Kumar, 1997; Maurya, 2010). Ashwagandha has been traditionally used as an herbal or metallic admixture that acts as rejuvenating and revitalizing agent. The species is distributed in Southeast Asia and also from the Mediterranean region to South Africa. Extracts from different plant parts like leaves, bark, stems, roots, and the entire plant are used for various therapeutic purposes including neurological, cardiovascular, gastric immunological conditions, and metabolic disorders such as diabetes (Mishra et al., 2000; Dar et al., 2017). The pharmacological effects of the species that have been thoroughly investigated over the years, chief phytoconstituents such as withanolide D and withaferin A, a group of steroidal lactones (Sharma et al., 2011) embarking much of the medicinal property. The other phytochemical constituents include steroidal lactones (glucosides-sitoinosides VII/VIII), cuscohygrine, tropine, alkaloids (withanine, somniferine, withananine, sominone, somnine, etc.), and saponins (Mishra et al., 2000).

Therapeutically Ashwagandha extract is used as an adaptogen, memory enhancer, aphrodisiac, energy tonic, and in the treatment of depression, hypertension, general debility, as anxiolytic, astringent, diuretic, narcotic, thermogenic, depurative, and stimulant, anthelmintic, anti-stress, anti-inflammatory, anti-carbuncle, in rheumatism, constipation, insomnia, leucoderma, nervous breakdown, goiter, leucorrhea, piles, and oligospermia (Agarwal et al., 1999; Machiah et al., 2006; Machiah and Gowda, 2006).

Ashwagandha extract is reported as a potent enhancer of cellular antioxidant mechanisms (Parihar et al., 2004) and exhibits a free radical scavenging activity. It is also reported to strengthen morphine-induced analgesia, averts the progress of morphine-induced rebound hyperalgesia (Orrù et al., 2014) and attenuates the development of tolerance to morphine's analgesic effects. The most likely mechanism involves multiple roles on neurotransmitters acting synergistically; it might block the GABA binding to its receptors as a result of an increase in chloride influx in the absence of GABA (Ruiu et al., 2013). Keeping that in mind, Gupta and Rana (2008) hypothesized that formulations of Ashwagandha extract might help in reducing ethanol withdrawal-induced anxiety and potentiate ethanol-induced anxiolysis (Gupta and Rana, 2008).

### *Silybum marianum* (L.) Gaertn

*Silybum marianum* (L.) Gaertn. (Asteraceae), commonly known as milk thistle, is an important annual/biannual plant growing to a height of 1.5 m long (Rambaldi et al., 2005). The species is native to the Mediterranean region; however, nowadays it is grown and cultivated around the world (Abenavoli et al., 2010). Traditionally, the plant was used as “galactogogue” (Ross, 2008). For more than 2000 years the plant has been used in the treatment of liver, kidney, spleen, headache, dyspepsia, eczema, migraine, psoriasis, and digestion disorders and gallbladder diseases (Gupta and Gupta, 2017; Tewari et al., 2017b). The species has antioxidant, antidiabetic, antihypertensive, antiatherosclerotic, and hypolipidemic properties that are useful in the treatment of liver and gallbladder disorders, including hepatitis, liver cirrhosis, and jaundice, and play a preventative role in cancer, neurodegenerative disorders such as Parkinson´s and Alzheimer´s diseases (Kren and Walterova, 2005; Bahmani et al., 2015; Tajmohammadi et al., 2018). Its herbal formulations are used against food poisoning, seasonal allergies, and several chemical and environmental toxins consumptions, i.e., alcohol intoxication and *Amanita phalloides* mushroom poisoning, acetaminophen, carbon tetrachloride, iron overload, phenylhydrazine, or bites and stings by snakes and insects (Abenavoli et al., 2010; Corchete; Kren and Walterova, 2005; Gupta and Gupta, 2017).

The above-mentioned pharmacological effects of milk thistle are derived from multiple bioactive compounds with potent biological properties. ‘Silymarin' that is basically a composite mixture of flavonolignans (flavanone derivative) obtained from fruits and seeds (achenes) of the plant, accounts for nearly 70-80% of the pharmacopeia and represents nearly 1.5-3% of the dry weight (Abenavoli et al., 2010; Tajmohammadi et al., 2018). The important major constituent present in silymarin is silybin (silibinin) that is a mixture of diastereoisomers, silybin A and B, accounting for nearly 50% of the extract. Other bioactive components present in silymarin are silychristin (about 20%), silydianin (about 10%), as well as isosilybin A and B (both approx. 5%). For several centuries milk thistle has been used as a natural remedy for a number of disorders of which a prominent one is ALD. A report of WHO (2012) states that of the total number of deaths globally due to liver cirrhosis, approximately 50% was caused by excessive and prolonged intake of alcohol (Hao et al., 2017). Out of the total cases of death globally alcohol leads to 1% of them (Masarone et al., 2016). A chronological series of events towards the progression of ALD includes alcoholic steatosis and steatohepatitis, fibrosis, and cirrhosis and lastly the development of hepatocyte carcinoma (Bataller and Brenner, 2005). ALD is a major cause of chronic liver injury, which results in liver fibrosis and cirrhosis, which is associated with the development of proinflammatory and profibrogenic cytokines, liver peroxidation, and ROS. Though, the pathogenesis of alcohol-induced organ damage is known, current therapies are not adequate and effective. Silymarin has gained in importance due to its cytoprotective property (Das and Mukherjee, 2012) and to the fact that upon intake, it concentrates within or near hepatocytes cells (Flora et al., 1998). Silymarin is also known to show competitive behavior with several biological toxins resulting into its blockade and thus preventing toxins penetration inside the hepatocyte cell, ultimately resulting into its protection. Apart from this, as ethanol metabolism is associated with amplified production of harmful ROS, silymarin by virtue of its potent antioxidant and scavenging property is known to effectively counteract these ROS species, including inhibiting lipid peroxidation and so it can be used as a supplement in the therapy of alcoholic liver cirrhosis (Saller et al., 2001; Corchete, 2008). Likewise, silymarin is also reported to stimulate nucleolar polymerase, an enzyme system controlling synthesis of ribosomal protein that in turn stimulates liver regeneration capability and new hepatocytes formation, therefore enhancing liver regenerative capacity (Boerth and Strong, 2002).

Despite these beneficial effects of silymarin, few clinical studies have been conducted over the years. Ferenci et al. (1989) studied effects of silymarin on 170 patients diagnosed with liver cirrhosis; out of these 92 patients were specifically diagnosed with alcoholic liver cirrhosis. Two groups of patients were delineated; one received oral administration of silymarin (i.e., 140 mg/day) three times a day while the control group was given placebo treatment for 2 years. It turned out that of the total number of deaths that occurred during the experiment the number in the placebo group was by twice higher (Ferenci et al., 1989). In another set of experiments, Vailati et al. (1993) studied alcoholic and viral chronic hepatitis patients using different doses of silymarin for 2 weeks. The doses of 160 mg/day were administered to 19 patients, of 240 mg/day to 17 patients and of 360 mg/day to 18 patients. The results highlight a significant decrease in hepatic biochemical profile of both ALT and gamma‐glutamyl transferase (GLT) levels as observed in the groups treated with 240 or 360 mg of silybin/day (Vailati et al., 1993). Feher et al. (1989) performed a 6-month double-blind liver functional test, involving serum, pro-collagen III and liver histology in 36 patients suffering from ALD. Liver functionality tests of the 17 ALD patients that were given 140 mg/day of silymarin for 6 months showed normalized functional behavior of serum bilirubin, AST and ALT, while a significant decrease in gamma-glutamyl transferase (GGT) and procollagen III was reported in the treated group as compared to the placebo group where only a decrease in GGT was observed, which was smaller than the treated group. Positive effects of silymarin were also reported on lymphocyte proliferation and lipid peroxidation as compared to the placebo group (Saller et al., 2001).

Das and Mukherjee (2012) studied the effectiveness of silymarin against ethanol-induced oxidative damage in the experimental mice. BALB/c 2–3 months mice with a body weight of 20–30 g were divided into four different groups. Group one was given 1.6 g/kg of ethanol, group two was exposed to 1.6 g/kg of ethanol plus 250 mg/kg of silybin, while the third group was fed with 250 mg/kg ethanol and 250 mg/kg ascorbic acid per day for 3 months, whereas the controlled group received isocaloric glucose solution. On histological and enzymatic analysis it was found that the levels of thiobarbituric acid and glutathione-S-transferase (GST) were significantly elevated in the blood hemolyzate biochemical profile analysis of the mice fed with ethanol. A noteworthy reduction in GSH and in several biochemical activities such as superoxide dismutase (SOD), catalase (CAT), glutathione reductase (GR), and glutathione peroxidase (GPx) was observed, while groups fed with silymarin counterchecked all the elevated parameters as seen in the ethanol group. Furthermore, silymarin drastically prevented the changes of molecular docks associated with ethanol consumption leading into several diseases such as interleukin (IL-4 & IL-10), TNF-α, vascular endothelial growth factor (VEGF-A), TGF-β1, and gamma interferon (IFN-γ) (Das and Mukherjee, 2012).

## Preclinical And Clinical Research Based On Ethnopharmacological Applications Targeting Alcohol Abuse

**Figure 2:**
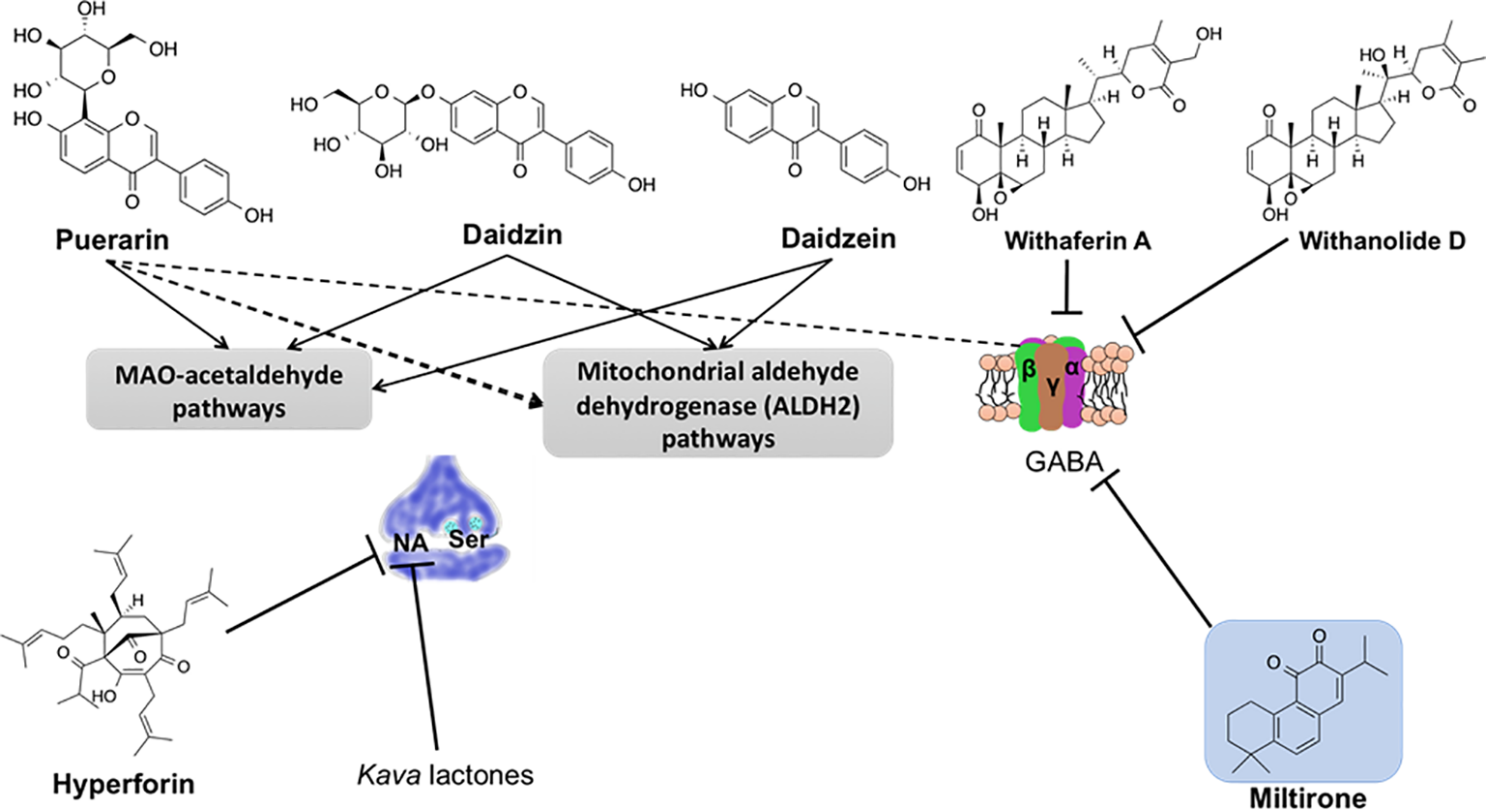
Possible mechanism of action of some of phytoconstituents in the context of counteracting alcohol abuse. Puerarin, daidzein, and daidzin decrease alcohol consumption by alterations in MAO-acetaldehyde pathways or mitochondrial ALDH2 pathways (Keung, 2003; Lukas et al., 2013); Withaferin A and withanolide D block GABA receptors binding and upsurges chloride influx in absence of GABA (Gupta and Rana, 2008; Lu et al., 2009; Ruiu et al., 2013); Kava lactones bind to multiple locations in the brain and interact with different neurotransmitters and significantly inhibit the uptake of noradrenaline, but not serotonin (Sällström Baum et al., 1998); Militirone is low-affinity ligand for central GABAA-BDZ–binding site, thus acting as a partial agonist and implying an anxiolytic effect (Lee et al., 1991).

A number of preclinical and clinical studies with natural products-based medicines have been performed to treat drug dependence, including alcoholism. Reports from some of the preclinical studies have shown that NR-ANX-C (standardized polyherbal formulation) consisting of extracts of *Ocimum tenuiflorum* L. (syn. *Ocimum sanctum* L.), *Withania somnifera* (L.) Dunal, *Camellia sinensis* (L.) Kuntze, *Zanthoxylum rhetsa* (Roxb.) DC., triphala (*Terminalia chebula* Retz., *Terminalia belerica* (Gaertn.) Roxb., and *Phyllanthus emblica* L. (syn. *Emblica officinalis*), and shilajit withdrawals ethanol induced anxiety behavior in rats (Nair et al., 2011), heightened ethanol-induced anxiolysis (Gupta and Rana, 2008), and weaken acquisition of oral ethanol administration under fixed and systemic increase in ratios (Peana et al., 2014). In addition to this, it decreased the deprivation effects and did promote the reinstatement state in ethanol-seeking behaviors in experimental rat model (de Wit and Stewart, 1981; Spina et al., 2015). Notably, the acquisition of ethanol-elicited mechanisms has been attributed to a number of cumulative neurological events involving receptors such as GABA_A_ (Chester and Cunningham, 1999) and GABA_B_ (Agabio and Colombo, 2014; Peana et al., 2014), dopamine (Spina et al., 2010), serotonin (Sellers et al., 1992; Koob, 2003), endogenous opioid receptor (Gianoulakis, 2009), and adenosine transmission (López-Cruz et al., 2013). Further, Gupta and Rana (2008) posited that the downregulation of GABA_A_ receptors or decrease in the GABAergic transmission may have been connected to alcohol withdrawal symptoms (Gupta and Rana, 2008). This in turn suggests that GABA mimetic and adaptogenic effect of Ashwagandha may further decrease the regulation of GABA_A_ receptor. The possible mechanisms of action of some phyto-constituents are presented in **Figure 2**.

The use of medicines based on natural products fits well with new trends in the treatment of drug dependence, such as alcoholism. **Tables 2** and **3** review the effects of a wide variety of extracts and some active plant constituents in animal models and clinical trials, respectively, in relation to alcohol dependence and abuse.

**Table 2:** Preclinical research based on ethnopharmacological applications targeting alcohol abuse.

Species	Common name	Plant extract or compound	Test model	Results	Reference
*Aesculus hippocastanum* L.	Horse Chestnut	Escins Ia, Ib, IIa, IIb, and IIIa	Male Wistar rats	Escins Ia, Ib, IIa, and IIb inhibit ethanol absorption.	(Yoshikawa et al., 1996b)
*Aralia elata* (Miq.) Seem.	Chinese angelica-tree, Japanese angelica-tree, and Korean angelica-tree	Oleanolic acid, 28-*O*-bisdesmosides, and oleanolic acid 3-*O*-monodesmosides	Male Wistar rats	Inhibitory effect on ethanol absorption.	(Yoshikawa et al., 1996a)
	Chinese angelica-tree, Japanese angelica-tree, and Korean angelica-tree	3-*O*-monodesmosides	Male Wistar rats	Elatoside A showed potent inhibitory activity on ethanol absorption.	(Yoshikawa et al., 1993)
*Buzui*	Buzui	Fruit of *Schisandra chinensis* (Turcz.) Baill.*, Terminalia chebula* Retz.*, Dark plum fruit* and *Crataegus pinnatifda* Bunge, Chicken's gizzard membrane and Silkworm excrement	Male pathogen-free (SPF) Kunming mice	Induces wakefulness and prevents acute alcohol intoxication, accelerates alcohol metabolism and thereby reduces oxidative damage.	(Chen et al., 2016)
*Camellia japonica* L.	Common camellia, Japanese camellia, Rose of Winter	Camellia saponins A1, A2, B1, B2, C1, and C2	Male Wistar rats	Camellia saponins B1, B2, C1, and C2 exhibit inhibitory ethanol absorption activity.	(Yoshikawa et al., 1996)
*Galanthus nivalis* L. and *Peganum harmala* L.	Snowdrop and Syrian rue	Galanthamine	Female Alko alcohol (AA) rats	Desoxypeganine–HCl reduces ethanol preference and intake while systemically increasing the dose concentration (10 and 30 mg/kg of the body weight). Desoxypeganine–HCl when applied in subcutaneous and intraperitoneal regions of the body leads to prominent reduction in ethanol preference and intake.	(Doetkotte et al., 2005)
*Ginkgo biloba* L.*, Mentha arvensis* L. *var. piperascens, Citrus deliciosa* Ten. *(syn. Citrus unshiu)* Blanco*, and Pueraria montana* var. *lobata* (Willd.) Maesen & S.M.Almeida ex Sanjappa & Predeep	-	Combined aqueous extracts (BHR)	Male Sprague-Dawley rats	BHR extract significantly reduces BALs and reduces area under curve (AUC) and C_max_ values in BHR treated rats at a dose concentration of 1 and 3 g/kg.	(Soo Shin et al., 2005)
*Glycine max* (L.) Merr.	Soybean	Milk	Male Sprague-Dawley rats	Demonstrates that soymilk products inhibit ethanol absorption and enhance ethanol metabolism in rats.	(Kano et al., 2002; Kano and Kubota, 2013)
*Hovenia dulcis* Thunb.	Korean raisin tree	Fruit extract	Mice	Reduces blood alcohol concentration by increasing the efficiency of ADH and glutathione-S-transferase (GST) activity and thus increases detoxification.	(An et al., 1999)
		Seed extract from China and Korea	Rats	Both extracts (crude and partitioned) accelerate the reducing rate of blood alcohol concentrations down to 1–2 h, compared to that of control.	(Kim et al., 2000)
		Ethanol and aqueous fruit extract	Rats	Reduces blood alcohol concentration by increasing the activity of ADH, ALDH, and GST activity and thus increases detoxification.	(Cha et al., 2004)
		Fruit water extract	Rats	Shows significant alcohol decrease in blood and hepatoprotective activity against CCl_4_-toxicity.	(Kim et al., 2006)
		Fruit water extract	Rats	The fruit extract (methanol and hot water extract) reduces acute alcohol toxicity and shows potent hepatoprotective activity against chemically, i.e., CCl_4,_ induced liver injury model.	(Kim et al., 2008)
		Dihydromyricetin (DHM)	Sprague-Dawley rats	Determines anti-alcoholic effects of DHM on animal models and put forward a major molecular target and cellular mechanism of DHM against alcohol dependence and intoxication.	(Shen et al., 2012)
*Hypericum perforatum* L.	St John's wort (SJW)	*Hypericum perforatum* extract (HPE)	cAA rats	*Hypericum* extract Ze 117 (Remotiv^®^) reduces EtOH intake in a selective manner thus revealing that the extract may be an interesting adjunct for the treatment of alcoholism.	(De Vry et al., 1999)
		*Hypericum perforatum* extract (HPE)	Marchigian Sardinian alcohol-preferring (msP) rats	Antidepressant-like effect of HPE extract in the force swimming test (FST) may be mediated by interaction of sigma receptors and to some extent by increased serotonergic neurotransmission.	(Panocka et al., 2000)
		*Hypericum perforatum* extracts (HPE)	Marchigian Sardinian alcohol-preferring (msP) rats	HPE noticeably reduces ethanol intake in msP rats, without affecting food intake.	(Perfumi et al., 1999)
		Methanolic extract (with 0.3% hypericin and 3.8% hyperforin) (HPE1) and CO_2_ extract (HPE2) with 24.33% hyperforin and very less hyperricin.	Marchigian Sardinian alcohol-preferring (msP) rats	HPE2 hinders ethanol intake more effectively than HPE1; higher HPE2 potency parallels the content of hyperforin, taking the role of hyperforin in reducing ethanol intake.	(Perfumi et al., 2001)
		*Hypericum perforatum* extracts (HPE)	Marchigian Sardinian alcohol-prefering (msP) rats	HPE inhibitory effects on ethanol intake are not mediated by GABA agonist actions.	(Perfumi et al., 2002)
		*Hypericum perforatum* CO_2_ Extract	Marchigian Sardinian alcohol-prefering (msP) rats	CO_2_ extract of *H. perforatum* and opiate receptor antagonists synergistically act to induce selective reduction of voluntary consumption of ethanol in alcohol-preferring rats.	(Perfumi et al., 2003)
		*Hypericum perforatum* extracts (HPE)	Fawn-hooded (FH) and high-alcohol drinking (HAD) rats	Demonstrates that acute or repeated oral administration of HPE produce dose-dependent reduction in alcohol intake in rats.	(Rezvani et al., 1999)
		*Hypericum perforatum* extracts (HPE)	Adult male C57BL/6J mice	Hyperforin contributes to observed reduction in alcohol intake.	(Wright et al., 2003)
*Jodina rhombifolia* (Hook. & Arn.) Reissek	Sombra de toro	Lyophilized aqueous extract (JRLE)	Male Wistar rats	Repeated administration of JRLE extract, noticeably reduce voluntary ethanol intake in male Wistar rats. This reduction in terms of consumption was of notable magnitude and remained stable during the 10-days of treatment.	(Teves et al., 2015)
NPI-028	NPI-028	Chinese herbal mixture: *Pueraria montana* var. *lobata* (Willd.) Maesen & S.M.Almeida ex Sanjappa & Predeep (syn. *Pueraria lobata*) (roots and leaves) and *Citrus* × *aurantium* L. (syn. *Citrus reticulata*) (fruit peel), *Panax ginseng* C.A.Mey. (leaves), *Glycyrrhiza uralensis* Fisch. ex DC. (roots), *Hovenia dulcis* Thunb. (seeds*), Silybum marianum* (L.) Gaertn. (seeds), and *Stevia rebaudiana* (Bertoni) Bertoni (leaves)	Rats and monkeys	Significantly reduces alcohol intake in alcohol-preferring (P) rats deprived of alcohol, suggesting that it might reduce desire for alcohol intake. However, NPI-028 did not produce a taste aversion to a novel saccharin solution, so it does not have a similar mechanism of action as that of naltrexone, the opiate antagonist. NPI-028 also selectively and chronically reduced alcohol intake in high alcohol drinking (HAD) rats, which are resistant to the effects of many other drugs. Finally, it was shown that NPI-028 dose-dependently reduced alcohol intake in a group of alcohol-preferring African green monkeys after intramuscular or oral administration.	(Overstreet et al., 1997)
			Alcohol-preferring P and Fawn-Hooded (FH) rats	NPI-028 was also effective in counteracting the increase in alcohol intake normally seen after a period of alcohol deprivation, both following the IP and following oral routes of administration.	(Overstreet et al., 1996)
*Panax ginseng* C.A.Mey.	Red ginseng	Red ginseng extract	Male Fischer rats	Rats plasma levels of ethanol are lowered when ethanol is administered orally along with ginseng than when administered singly, but the previous one has no effect on plasma levels of ethanol administered intraperitneally.	(Lee et al., 1993)
			Male Fischer rats	Rats ethanol plasma levels are lowered by (20%) when alcohol and red ginseng extract were orally administered than when only alcohol was administered.	(Kwak and Joo, 1980)
			Rats	Increased the rate of oxidation of ethanol in alcohol-fed rats.	(Joo et al., 1982)
*Panax guingnefolium* L.	Ginseng	Total saponin from steam and leaves	Rats	Inhibition of gastro-intestinal tract absorption of ethanol.	(Ma et al., 1992)
*Passiflora edulis* Sims	Passion flower	Benzoflavone moiety extract	Swiss albino mice	In Chronic and acute administrations the benzoflavone moiety significantly prevented the alcohol withdrawal expression and decreased ethanol induced anxiety behavior in mice.	(Dhawan et al., 2002)
*Piper caldense* C. DC.	Pimenta-darda	Hydroalcoholic extract of leaves	Male Wistar rats	Showed a significant effect, reducing alcohol consumption compared to the control group.	(Pereira et al., 2015)
*Pueraria montana* var. *lobata* (Willd.) Maesen & S.M.Almeida ex Sanjappa & Predeep (syn. *Pueraria lobata* (Willd.) Ohwi)	*Radix puerariae* (kudzu)	Daidzin and diazein	Syrian Golden hamsters	Daidzin and daidzein, at doses of 150 and 230 mg/kg suppressed ethanol intake by >50%. However, the above treatment did not significantly affect the body weight and water or food intake.	(Keung and Vallee, 1993a)
		Daidzin	Syrian golden hamsters	Daidzin treatment at a dose of 150 mg/kg per day (i.p. for 6 days) significantly suppresses voluntary ethanol intake by ≈70% in golden hamster but when its ability to inhibit acetaldehyde metabolism *in vivo* was tested, plasma acetaldehyde metabolism was not affected at all. Also Daidzin, effectively suppressed golden hamster liver mitochondria-catalyzed acetaldehyde oxidation with an IC_50_ value of 0.4 µM, which is substantially lower than the daidzin concentration (70 μM) found in the liver mitochondria of daidzin-treated hamsters.	(Keung et al., 1995)
		Daidzin	Male Wistar rats	Daidzin decreased sweetened ethanol consumption more than it did starch consumption. Changes in consumption were dose dependent, and differences in ethanol and food consumption increased slightly (but significantly) as dose increased.	(Heyman et al., 1996)
		Kudzu Root Extract (KRE)	Adult male Sprague–Dawley (SD) rats	Daidzin inhibits ALDH-2 and suppresses heavy drinking in rodents. Decreased drinking due to ALDH-2 inhibition is attributed to aversive properties of acetaldehyde accumulated during alcohol consumption.	(Arolfo et al., 2009)
		Kudzu Root Extract (KRE)	Alcohol-preferring (P) rats	A daily 50 mg/kg dose of puerarin (PU) caused approximately 50% suppression in alcohol intake, but did not affect body weight and food and total fluid intake in P rats receiving “free choice” of water and 15% ethanol. PU feeding transiently suppressed alcohol intake and abolished withdrawal symptoms at a time when alcohol intake had returned to the control level.	(Benlhabib et al., 2004b)
		Kudzu Root Extract (KRE)	Alcohol preferring (P) rats	A daily dose of 50 mg/kg of puerarin (PU) caused approximately 50% suppression in alcohol intake, but did not affect body weight and food and total fluid intake in P rats receiving “free choice” of water and 15% ethanol. PU feeding transiently suppressed alcohol intake and abolished withdrawal symptoms at a time when alcohol intake had returned to the control level.	(Benlhabib et al., 2004a)
		Ethanol extract	Male Wistar rats	Daidzin delayed and decreased peak blood alcohol concentration (BAC) level after ethanol intake. When ethanol (40% solution, 3 g/kg of body weight) was given to fasted rats intragastrically, BAC peaked at 30 min after alcohol ingestion and reached 1.77 ± 0.14 mg/mL. But when daidzin (30 mg/kg) was mixed with the ethanol solution and given to animals intragastrically, BAC was found to peak at 90 min after alcohol ingestion and reached only 1.20 ± 0.30 mg/ml.	(Xie et al., 1994)
		*Flos puerariae* lobatae water extract (FPE)	Male Sprague-Dawley rats and male BALB/C mice	FPE and its active ingredient puerarin have preventive effects on alcoholism-related disorders. Puerarin pretreatment, but not post-treatment, can reverse the changes of GABA_A_R subunit expression and increase ADH activity in alcoholism models.	(Zhang et al., 2010)
		Puerariae Flos isoflavonoid fraction (PF-IF)	mice	blood alcohol and acetaldehyde concentrations decreased more after the treatment	(Niiho et al., 1989)
		daidzin, daidzein and puerarin	Alcohol preferring (P) rats	suppressing the appetite for alcohol when taken orally	(Lin et al., 1996)
*Pyrus pyrifolia* (Burm.f.) Nakai (*syn. Pyrus pyrifolia cv. Shingo*)	Korean Pear	Korean Pear extract	ALDH2 normal (C57BL/6) and deficient (ALDH2 -/-) male mice	Pear extract stimulated both ADH and ALDH activities by 2∼3 in vivo and 1.3 fold in *in vitro* studies. The pharmacokinetic data (i.e., AUCα and C_max_) showed that the pear extract decreased the alcohol level in blood regardless of ALDH2 genotype and increased the acetaldehyde level in blood in Aldh2 deficient mice but not in ALDH2 normal mice.	(Lee et al., 2012)
*Sedum rosea* (L.) Scop. (syn. *Rhodiola rosea* L.)	Rhodiola (golden root)	Salidroside	Male Wistar rats	Indicates that salidroside at a dose of 45 mg/kg inhibited the development of tolerance to the hypothermic effect of ethanol. Observed inhibition of tolerance to the sedative effect of ethanol seems to be associated with salidroside influence on the CNS.	(Szulc et al., 2018)
*Salvia miltiorrhiza* Bunge	“Danshen” or “Tanshen”	Methanol extract	Sardinian alcohol-preferring (sP) rats	Effect due to its ability to alter ethanol absorption from the gastrointestinal tract. It reduced voluntary alcohol intake, and decreased BALs by approximately 60%.	(Colombo et al., 1999)
		*S. miltiorrhiza* extracts, differing in miltirone content (0, 2, 3, and 7%)	Sandinian alcohol-preferring (sP) rats	Alcohol intake was positively and significantly correlated with miltirone content of the extracts. *S. miltiorrhiza* extracts, miltirone markedly reduced BALs when alcohol was administered i.g. but not i.p., suggesting that miltirone hampered alcohol absorption from the gastrointestinal system.	(Colombo et al., 2006)
		Standardized extract (IDN 5082)	Sardinian alcohol-preferring (sP) rats	Dose-dependently delayed acquisition of alcohol-drinking behavior.	(Brunetti et al., 2003)
		Standardized extract (IDN 5082)	Sardinian alcohol-preferring (sP) rats	Prevents the development of the alcohol deprivationeffect (ADE). The acute, intragastric administration of 25, 50, and 100 mg/kg resulted in the complete suppression of the extra amount of alcohol consumed during the first hour of re-access to alcohol after 7 days of deprivation. The results indicated that IDN 5082 might possess antirelapse properties.	(Serra et al., 2003)
		Ethanol extract	Sardinian alcohol-preferring (sP) rats	A significant and specific reduction in alcohol intake was recorded only in rats treated with the combination of Polysorbate 80 plus the *S. miltiorrhiza* extract.	(Vacca et al., 2003)
*Salvia przewalskii* Maxim.	Red sage	Hairy roots and callus cultures extract	Male Warsaw High Preferring Wistar rats (WHP)	Significantly reduced alcohol intake in alcohol-dependent animals. This activity was correlated with the content of tanshinones (cryptotanshinone) in callus extract, but not with phenolic acids.	(Gryszczynska et al., 2015)
SKV	Asuuam	Fermentation of cane sugar, raisins, and water and 12 herbal ingredients: *Piper nigrum* L. seeds, *Piper longum* L. seeds, *Santalum album* L. heartwood, *Pterocurpus santalinus* L.f. heartwood, *Nardostachys* *jatamansi* (D.Don) DC. roots, *Symplocos racemosa* Roxb. bark, *Chrysopogon zizanioides* (L.) Roberty (syn. *Andropogen muricatus*) roots, *Elettaria cardamomum* (L.) Maton seeds, *Berberis aristata* DC. root/bark/- stem, *Plumbago zeylanica* L. roots and *Cyprus rotundus* L. tubers, *Woodfordia fruticosa* (L.) Kurz (syn. *Woodfordia floribunda*) flowers.	Adult albino male rats	Brought down voluntary alcohol ingestion and increased food intake.	(Shanmugasundaram and Shanmugasundaram, 1986)
			Adult albino male rats	Rats on SKV therapy with free access to 15% ethanol showed a marked reduction in voluntary ethanol intake.	(Shanmugasundaram et al., 1986)
*Strychnos nux-vomica* L.	Nux vomica	Mother tincture (MT), Nux 30c, and its principal alkaloid, strychnine	Albino rats of the Charles Foster strain	Nux MT and Nux 30c could reduce ethanol intake in rats. The altered solution structure of Nux 30c is thought to mimic Nux MT and produce ethanol aversion in rats.	(Sukul et al., 2001)
*Tabernathe iboga* Baill.	Iboga	Ibogaine	Sprague-Dawley rats	Reduces volitional alcohol consumption in alcohol-preferring rats. Exerted its anti-craving effects on voluntary alcohol intake by interacting with the brain parts involved in stimulating dopaminergic and serotonergic systems.	(Glick et al., 1991)
			Fawn-Hooded rats	Ibogaine when injected into different regions of the body, i.e., intraperitoneal or intragastric but not subcutaneous, can significantly reduce alcohol intake without an effect on blood alcohol concentrations or food intake.	(Rezvani et al., 1995b)
		Noribogaine	P and Fawn-Hooded rats	Significantly suppressed alcohol intake in alcohol preferring rats.	(Rezvani et al., 1995a)
		18-Methoxycoronaridine (18-MC)	Adult male alcohol-preferring rats	Significantly and dose-dependently attenuated alcohol consumption and preference and commensurately increased water intake.	(Rezvani et al., 1997)
*Thymus vulgaris* L.	Thyme	Water extract	Male Albino mice	Detoxifying and antioxidant effects.	(Shati and Elsaid, 2009)
*Withania somnifera* (L.) Dunal	Indian ginseng	Roots extract (WSE)	Adult male Wistar rats	WSE reduced the acquisition, maintenance breakpoint of ethanol self-administration and reinstatement of ethanol-seeking behaviors. The GABA_B_ receptor antagonist, phaclofen, counteracted the ability of WSE to impair the maintenance of ethanol self-administration.	(Peana et al., 2014)
*Zingiber officinale* Roscoe	Ginger	Water extract	Male Albino mice	Significant increase in NO and malondialdehyde level in liver and brain and a decrease in the total antioxidant capacity and GPx activity in alcoholic group. The extract has potent detoxifying and antioxidant effects.	(Shati and Elsaid, 2009)

**Table 3:** Clinical research based on ethnopharmacological applications targeting alcohol abuse.

Species	Common name	Plant extract or compound	Model	Results	Reference
*Hypericum perforatum* L.	St John's Wort (SJW)	*Hypericum* herbal infusion	Human	*Hypericum* herbal infusion was used in combination with rational psychotherapy of depressive manifestations in 57 outpatients with alcoholism and concomitant diseases of digestive organs. Duration of treatment was 2 months (1 glass 4–5 times daily). This treatment in combination with rational psychotherapy proved effective.	(Krylov and Ibatov, 1993)
*Jiejiu Jiedu*	Jiejiu Jiedu	Jiejiu Jiedu decoction *Coptis chinensis* Franch., *Phellodendron chinense* C.K. Schneid., *Angelica sinensis* (Oliv.) Diels, *Aconitum carmichaeli* Debeaux, *Actaea heracleifolia* (Kom.) J.Compton, *Bupleurum chinense* DC., *Aucklandia costus* Falc., *Pinellia ternata* (Thunb.) Makino, *Ophiopogon japonicus* (Thunb.) Ker Gawl., *Schisandra chinensis* (Turcz.) Baill., and *Glycyrrhiza uralensis* Fisch. ex DC.,	Human	Antidipsotropic action of Jiejiu Jiedu decoction was as good as furazolidone.	(Cao et al., 2007)
*Lophophora williamsii* (Lem.) J.M.Coult.	Peyote	Peyote button	Human	Ritualistic use of Peyote to a properly structured psychotherapeutic session has been demonstrated to be an effective technique for treating alcoholics.	(Albaugh and Anderson, 1974)
NPI-031	Alkontrol-herbal™	Standardized Kudzu extract (NPI-031)	Human	Significantly reduced the number of drinks consumed each week by 34–57%, reduced the number of heavy drinking days, and significantly increased the percent of abstinent days and the number of consecutive days of abstinence.	(Lukas et al., 2013)
			Human	Currently underdevelopment	ClinicalTrials.gov Identifier: NCT03099590
	Kudzu	Extract	Male and female “heavy” alcohol drinkers	Significant reduction in the number of beers consumed that was paralleled by an increase in the number of sips and the time to consume each beer and a decrease in the volume of each sip.	(Lukas et al., 2005)
*Panax ginseng* C.A.Mey.	Ginseng	Water extract	Human	Ingestion of ginseng along with alcohol accelerates blood alcohol clearance and may render clinical applications in the treatment of alcoholic patients and help alleviate many detrimental effects caused by acute ethanol intoxication.	(Lee et al., 1987)
*Pediculus melo*	Musk melon base	*P. melo* wine	Human	Significant decline in alcohol intake after taking *P. melo* wine.	(Dou et al., 2003)
		Guadi capsule, containing 0.2 g *P.* *melo*	Human	Confirmed the study of Wang and highlighted the usage of *P. melo* with fewer side effects than apomorphine.	(Shang et al., 2005)
*Psilocybe mexicana* (Fungi)	Philosopher's stones	Psilocybin	Volunteers with DSM-IV alcohol	Abstinence did not increase significantly in the first 4 weeks of treatment (when participants had not yet received psilocybin), but increased significantly following psilocybin administration (p < 0.05). Gains were largely maintained at follow-up to 36 weeks. The intensity of effects in the first psilocybin session (at week 4) strongly predicted change in drinking during weeks 5–8 (r = 0.76 to r = 0.89) and also predicted decreases in craving and increases in abstinence self-efficacy during week 5.	(Bogenschutz et al., 2015)
*Pueraria montana* var. *lobata* (Willd.) Maesen & S.M.Almeida ex Sanjappa & Predeep (syn. *Pueraria lobata* (Willd.) Ohwi)	Kudzu	Kudzu root extract	Human	Appeared to be no better than placebo in reducing the craving for alcohol or promoting sobriety.	(Shebek and Rindone, 2000)
		Kudzu extract	Human	Reduces alcohol consumption in a binge drinking paradigm.	(Penetar et al., 2015)
	Kudzu (Puerariae Flos)	Dried flower extracts	Human	Probably promotes the elimination of blood acetaldehyde in humans and clinically. There might be a modest stimulatory effect of *P. thomsonii* on the elimination of blood acetaldehyde, may passively mitigate acetaldehyde toxicity symptoms, such as flushing, palpitation, headache, etc., associated with excessive alcohol intake.	(Yamazaki et al., 2002)
*Wendan* decoction	*Wendan* decoction (WDD)	WDD is typically composed of *Pinellia ternata* (Thunb.) Makino, *Phyllostachys nigra* var. *henonis* (Mitford) Rendle, *Citrus* × *aurantium* L., *Wolfiporia extensa*, *Zingiber officinale* Roscoe, *Ziziphus jujuba* Mill. and *Glycyrrhiza uralensis* Fisch. ex DC.	Human	Wendan decoction (500 ml, bid) was effective in treating alcohol dependence patients (overall effective rate: 83.3%).	(Qu and Wang, 2008)

Apart from the above studies, numerous plants are utilized in folklore medicine and as such are thoroughly investigated for their use in prevention or treatment of ethanol-induced liver injury. Some of these natural products like taraxasterol exhibit their protective potential against ethanol-induced liver damage because they regulate different signaling pathways like NF-*κ*B and CYP2E1/Nrf2/HO-1 in mice models (Xu et al., 2018). Studies also showed that *Monolluma quadrangula* (Forssk.) Plowes, *Geranium schiedeanum* Schltdl., and *Phyllanthus emblica* L. are also effective in this respect (Ibrahim et al., 2015; Madrigal-Santillán et al., 2015; Chaphalkar et al., 2017). Recently a number of reviews have been published on the topic (Guan et al., 2018; Singh et al., 2018).

## Conclusions And Outlook

Alcohol abuse and dependence is one of the most important public health problems worldwide. Over time, regular usage of substances such as alcohol, opioids, cigarettes, and tobacco has resulted in a habitual behavioral intake. Rehabilitation and discontinuation of these substance addictions remain a challenging task of research. At present efforts are focusing on the development of low-toxicity and high-efficiency natural remedies. Although the modern pharmacological approaches are known to play a key role in achieving complete alcohol abstinence and preventing relapse, their efficacy is still limited, accompanied with a great deal of side effects, tolerance development, and sensitization or dependence to such drugs (Addolorato et al., 2005a; Addolorato et al. 2005b; Uzbay, 2008). Taking this into account, search for an alternative and new psychotherapeutic medication from natural sources was emphasized for anti-addiction therapies. The extracts from *Hypericum perforatum*, *Puereria montana* var. *lobata*, *Withania somnifera*, *Panax ginseng*, *Macropiper methysticum*, *Salvia miltiorrhiza*, *Thunbergia laurifolia*, *Tabernanthe iboga*, etc., have demonstrated potent antidipsotropic effects in alcohol preferring or alcohol-fed rats (Lin et al., 1996; Lin and Li, 1998; Overstreet et al., 2003; Rezvani et al., 2003). Similarly extracts from *P. tenuifolia, T. laurifolia,* and *Simplocos racemosa* have been found to inhibit cocaine-craving behavior in rats (Chung et al., 2002, Thongsaard and Marsden, 2002). Extracts from *P. ginseng* and *Corydalis yanhusuo* may be clinically useful for the prevention of opioids abuse and to prevent relapse to chronic drug dependence. Sinomenine, an alkaloid from *Sinomenium acutum* (Thunb.) Rehder & E.H.Wilson, has been shown to have preventive and curative effects of opioid dependence. Rhynchophylline an alkaloid from *Uncaria rhynchophylla* (Miq.) Miq. ex Havil. is reported to have positive effects on methamphetamine and ketamine addiction. Likewise, L-Stepholidine, an alkaloid extract of the Chinese herb *Stephania intermedia* H.S. Lo, helps to control morphine-preference and induces reinstatement (Zhu et al., 2017).

Considering the limitations of the available pharmacotherapeutic agents, herbal remedies may provide an alternative. Herbal extracts and constituents with demonstrable psychotherapeutic effects in animal models deserve further clinical trials and evaluation. Further, the use of such natural formulations is still in its infancy stage. Further clinical and behavioral studies of herbal remedies might provide a unique opportunity for the development of new pharmacotherapies for alcohol withdrawal symptoms and prevention of relapse.

## Author Contributions

LS, TJ, DT, JE, AM, and AA drafted and conceived the manuscript. All authors revised and approved the final version.

## Conflict of Interest

Author NT was employed by company NTZ Lab Ltd.

The remaining authors declare that the research was conducted in the absence of any commercial or financial relationships that could be construed as a potential conflict of interest.

## Abbreviations

AA: Alko Alcohol
ADE: alcohol deprivation effect
ADH: alcohol dehydrogenase enzyme
AIDS: acquired immunodeficiency syndrome
ALD: alcoholic liver disease
ALDH: aldehyde dehydrogenase
ALF: acute liver failure
ALT: alanine aminotransferase
AST: aspartate aminotransferase
ATP: adenosine triphosphate
AUC: area under curve
AUD: alcohol use disorder
BAL: blood alcohol level
BCA: blood alcohol concentration
BCE: Before the Common Era
BDZ: benzodiazepine
CAT: catalase
CGX: Chunggan extract
CLD: chronic liver disease
CNS: central nervous system
CRH: corticotropin releasing factor
CTGF: connective tissue growth factor
CYP2E1: Cytochrome P450 2E1
D1: dopamine receptor 1
D2: dopamine receptor 2
DHM: dihydromyricetin
DA: dopamine
DOPAC: 3,4-dihydroxyphenylacetic acid
DOPAL: dihydroxyphenylacetic acid
FH: Fawn-Hooded
FOS: c-fos protein
FST: force swimming test
GABA: gamma-aminobutyric acid
GGT: gamma-glutamyl transferase
GLA: ?-linolenic acid
GPx: glutathione peroxidase
GSH: glutathione-S-transferase
GST: glutathione-S-transferase
GR: glutathione reductase
Had: high alcohol-drinking
HIV: human immunodeficiency virus
HSC: hepatic stellate cells
L-THP: Lev-tetrahydropalmatine
IFN-γ: gamma interferon
IL-4: interleukin 4
IL-10: interleukin 10
MAO: monoamine oxidase
MAPs: MITOGEN-activated proteins
msP: Marchigian Sardinian
NA: noradrenaline
NAFLD: non-alcoholic fatty liver disease
NEFAs: non-esterified fatty acids
Nrf2: nuclear factor erythroid 2–related factor 2
NO: nitric oxide
OR: odds ratio
P: alcohol-preferring
PDGF-β: platelet-derived growth factor-beta
PGE1: prostaglandins E1
PU: puerarin
ROS: reactive oxygen species
RTI: road traffic injury
Ser: serotonin
SJW: St. John’s wort
sP: Sardinian alcohol-preferring
SOD: superoxide dismutase
TGF-β: transforming growth factor-beta
THH: tetrahydroharmine
TNFα: tumor necrosis factor-alpha
TCM: traditional Chinese medicine
VACS: Veterans Aging Cohort Study
VEGF-A: vascular endothelial growth factor
WHO: World Health Organization
XJL: Natural Pharmacia International [NPI] preparation #28
18-MC: 18-methoxycoronaridine
5-HIAL: 5-hydroxyindole 3-acetaldehyde
5-HIAAA: 5-hydroxyindole-3-acetic acid
